# Supplementary material for: Rectifying disorder of extracellular matrix to suppress urethral stricture by protein nanofilm-controlled drug delivery from urinary catheter
Source: Nat Commun. 2023 May 17;14:2816. doi: 10.1038/s41467-023-38282-2 (PMC10192346; doi:10.1038/s41467-023-38282-2)
Supplement: Supplementary file 1 — Supplementary information [file 41467_2023_38282_MOESM1_ESM.pdf]

Supplementary Information

Rectifying disorder of extracellular matrix to suppress urethral stricture by protein nanofilm-controlled drug delivery from urinary catheter

Juanhua Tian[1], Delai Fu[1], Yongchun Liu[2], Yibing Guan[3], Shuting Miao[2], Yuquan Xue[1], Ke Chen[4], Shanlong Huang[1], Yanfeng Zhang[5], Li Xue[1], Tie Chong[1*], Peng Yang[2, 6, 7*]

*** Corresponding author: Peng Yang (email: yangpeng@snnu.edu.cn), Tie Chong (email: chongtie@126.com)**

1. Department of Urology, The Second Affiliated Hospital of Xi'an Jiaotong University, West Five Road, No. 157, Xi'an 710004, China.

2. Key Laboratory of Applied Surface and Colloid Chemistry, Ministry of Education, School of Chemistry and Chemical Engineering, Shaanxi Normal University, Xi'an 710119, China.

3. Department of Urological surgery, The First Affiliated Hospital of Zhengzhou University, Zhengzhou, Henan Province, China.

4. Key Laboratory of Bio-Inspired Smart Interfacial Science and Technology of Ministry of Education, School of Chemistry, Beihang University (BUAA), Beijing 100191, China.

5. School of Chemistry, Xi'an Jiaotong University, Xi'an, 710049, China.

6. International Joint Research Center on Functional Fiber and Soft Smart Textile, School of Chemistry and Chemical Engineering, Shaanxi Normal University, Xi'an 710119, China.

7. Xi'an Key Laboratory of Polymeric Soft Matter, School of Chemistry and Chemical Engineering, Shaanxi Normal University, Xi'an 710119, China.

Supplementary Information Content

Supplementary Methods
Supplementary Figures (1-47)
Supplementary Tables (1-5)
Supplementary References

Supplementary Methods.

Entrapment efficiency determination for drug in the delivery system.

The sustained-release catheters were soaked in acetonitrile solution and vigorously stirred for 4 h at room temperature. The solution was centrifuged at 10000 3354×g for 10 min. Then the supernatant solution was detected by a UV-spectrometer to determine the amount of rapamycin encapsulated in the drug delivery system. The entrapment efficiency was calculated by using the following formula:

Entrapment efficiency $= \frac{W}{W_0} \times 100\%$

where W_0 represents the initially added amount of drug and W represents the amount of drug entrapped in the drug delivery system.

Rapamycin measurement in whole blood.

The analysis of rapamycin concentration in whole blood was performed using a previously published method with a slight adaption[1]. Briefly, after adding 50 μL of fresh whole blood to 200 μL of methanol, the vials were vortexed for 5 min and centrifuged at 4,360.2×g for 10 min. The supernatant was injected and analysed with LC-MS/MS using a Thermo Fisher Scientific (Waltham, USA) triple quadrupole Quantum Access LC-MS/MS system. Data acquisition and data processing were performed using Xcalibur software version.

Immunostaining.

All paraffin-embedded tissue was cut into 5 μm thick continuous sections. Tissue sections were deparaffinized, rehydrated, antigen retrieval, and quenched in 3% hydrogen peroxide. After blocking with 5% BSA, the slides were incubated with primary antibody at 4ºC overnight, and then incubated with secondary horseradish peroxidase (HRP)-labelled rabbit/mouse anti-goat IgG antibodies (Zhongshan Corp.). The sections were visualized with a 3,3'-diaminobenzidine kit (DAB, Zhongshan Corp.) and counterstained with haematoxylin. The data was analyzed by ImageJ v1.8.0. Primary antibodies used in the staining include CD68 Mouse monoclonal antibody (abcam, ab955, 1:3000). MMP1 Rabbit polyclonal antibody (Proteintech, 10731-2-AP, 1:200) and anti-alpha smooth muscle actin monoclonal antibody (abcam, ab7817, 1:200).

Immunofluorescence.

Primary rat urethral fibroblasts were seeded on coverslips in 24-well plates. After the cells attached, the coverslips with attached cells were taken out and fixed with 4% paraformaldehyde for 15 min at room temperature, followed by permeabilization with 0.3% Triton for 15 min. After blocking with 5% BSA at room temperature for 1 h, the primary antibody was dropped onto the coverslips and incubated overnight at 4°C. Then, they were incubated with Alexa Fluor 488-labled Goat Anti-Rabbit IgG (1:500, A0423, Beyotime Institute of Biotechnology, China) at room temperature for 40 min, and the nuclei were stained with 4,6-diamino-2-phenylindole (DAPI, C1002, Beyotime Institute of Biotechnology, China).

For paraffin-embedded tissue sections, antigen retrieval was performed with citrate buffer before permeabilization. Antibodies against CD45 Rabbit Polyclonal antibody (1:500, Gene Tex, GTX116018), Vimentin (1:500, D21H3, CST, USA), collagen I (1:400, 14695-1-AP, Proteintech, China) and collagen III (1:400, ab7778, abcam, UK) were diluted with 1% bovine serum albumin in PBS. The image was taken using a confocal laser scanning microscope.

Supplementary Figures.

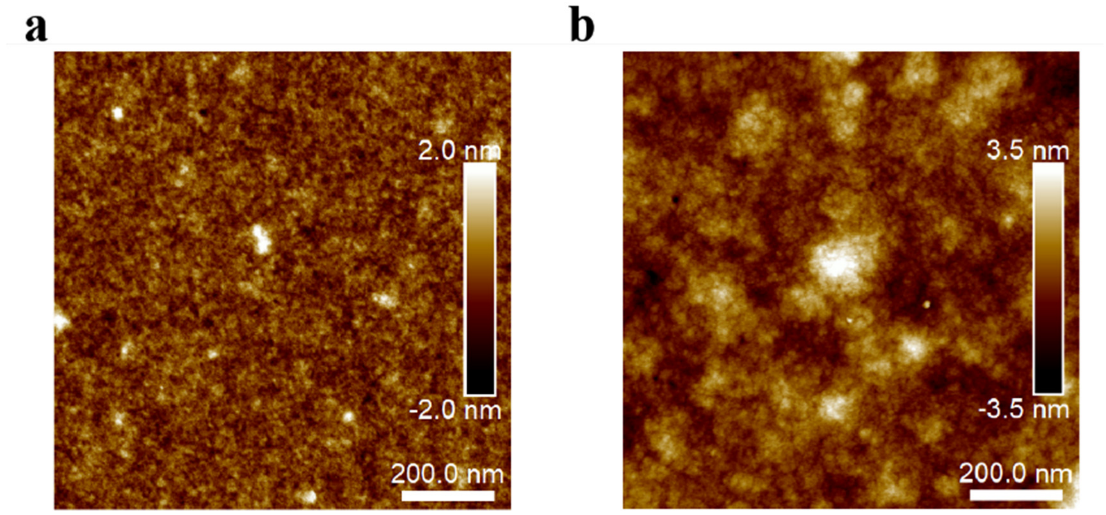

Supplementary Figure 1. The AFM images showing the PTB@SA coating morphology at different reaction time. (**a**) 5 min; (**b**) 10 min.

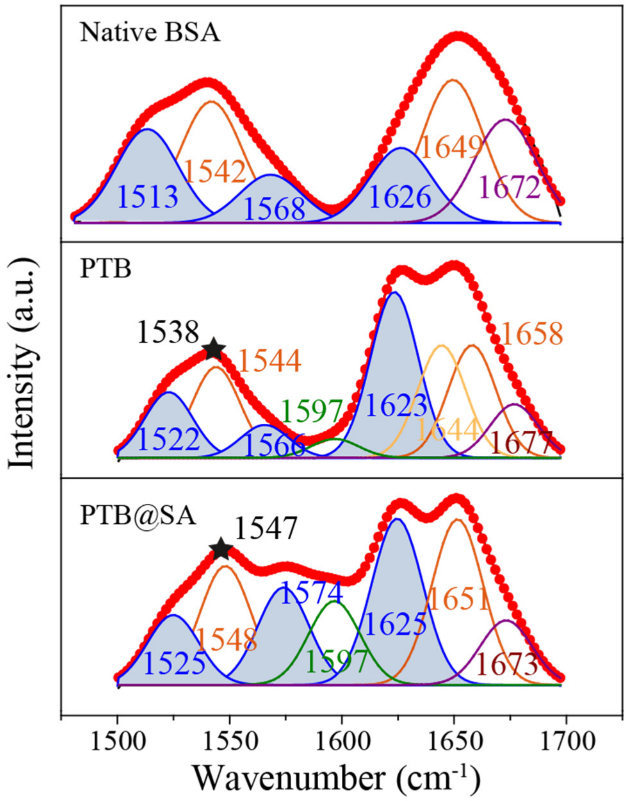

Supplementary Figure 2. FTIR peaks of amide I and amide II of native BSA, the pure PTB or the hybrid PTB@SA nanofilm. The blue area and asterisk indicate the deconvoluted β-sheet band and the main peak of amide II, respectively.

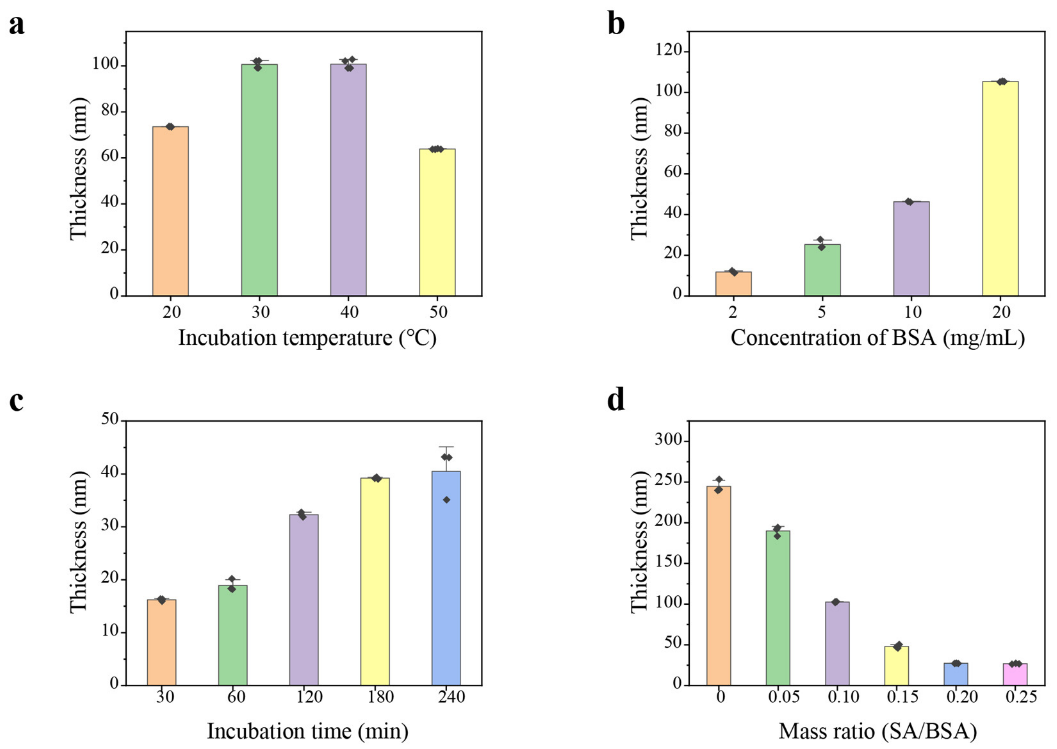

Supplementary Figure 3. The thickness of the hybrid PTB@SA nanofilm controlled by the incubation temperature (a), BSA concentration (b), incubation time (c) and SA addition ratio (d). (**a**). The final concentrations of BSA, TCEP, and SA in the phase transition solution for manufacturing the coating were 8 mg/mL, 20 mM and 0.8 mg/mL, respectively. The incubation time was 3 h, and the reaction temperature varied from 20°C to 30°C. n=4 independent samples per group. (**b**). For a given mass ratio of SA to BSA (0.10), the final concentration of BSA in the phase transition solution was ranged from 0.8 to 8 mg/mL, and the reaction temperature and time were 30°C and 3h, respectively. n=3 independent samples per group. (**c**). The final concentrations of BSA, TCEP, and SA in the phase transition solution used to prepare the coating were 4 mg/mL, 20 mM, and 0.4 mg/mL, respectively, and the incubation time at 30°C varied from 30 to 240 min. n=3 independent samples per group. (**d**). The final concentrations of BSA and TCEP are 8 mg/mL and 20 mM, and the mass ratio of SA to BSA in the phase transition solution varied from 0.05 to 0.25. The reaction temperature and time were 30°C and 3 h, respectively. n=3 independent samples per group. All data are presented as the Mean ± S.D.

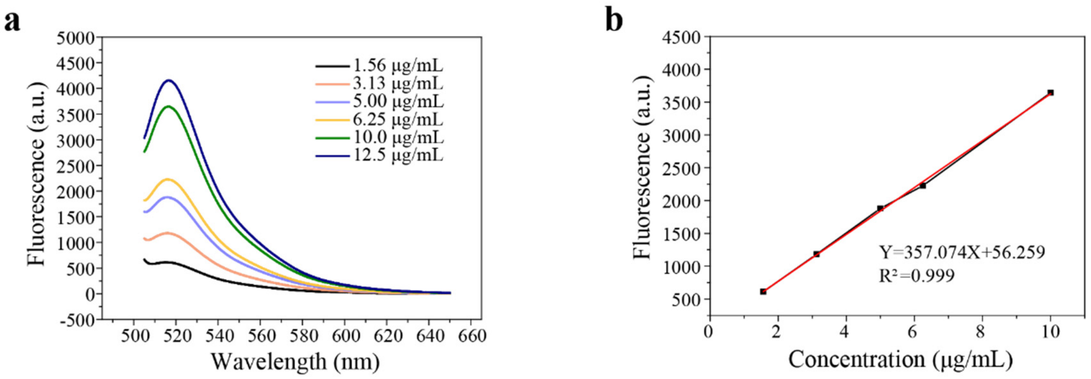

Supplementary Figure 4. Encapsulation density of SA in hybrid nanofilms. (**a**) The fluorescent spectra of 5(6) -aminofluorescein-labeled SA in 2 M NaOH at different concentrations. (**b**) Standard curve and regression equation of 5(6) -aminofluorescein-labeled SA in 2 M NaOH.

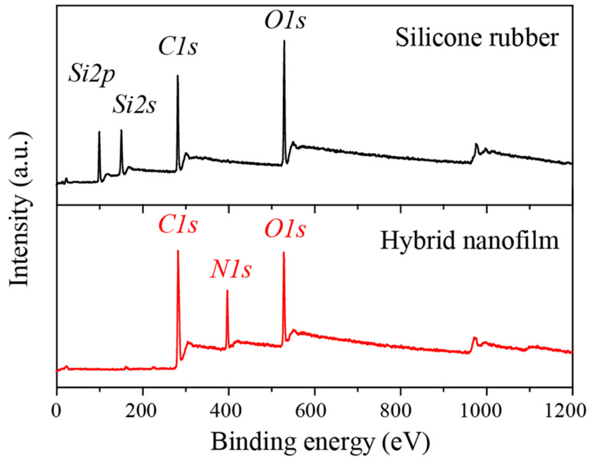

Supplementary Figure 5. XPS spectra of pristine silicone rubber (SR) and the hybrid PTB@SA nanofilm coated SR.

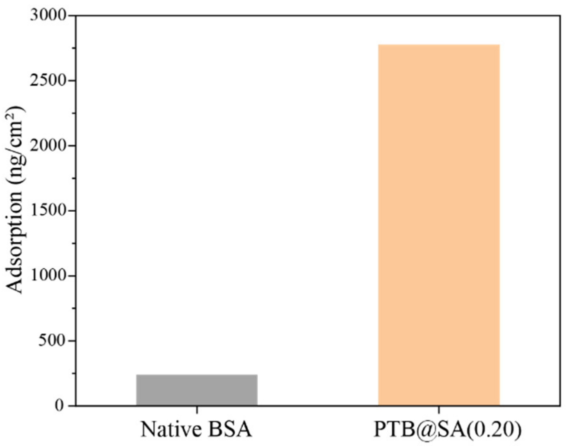

Supplementary Figure 6. The adsorption amount of native BSA and PTB@SA(0.20) on Au chips.

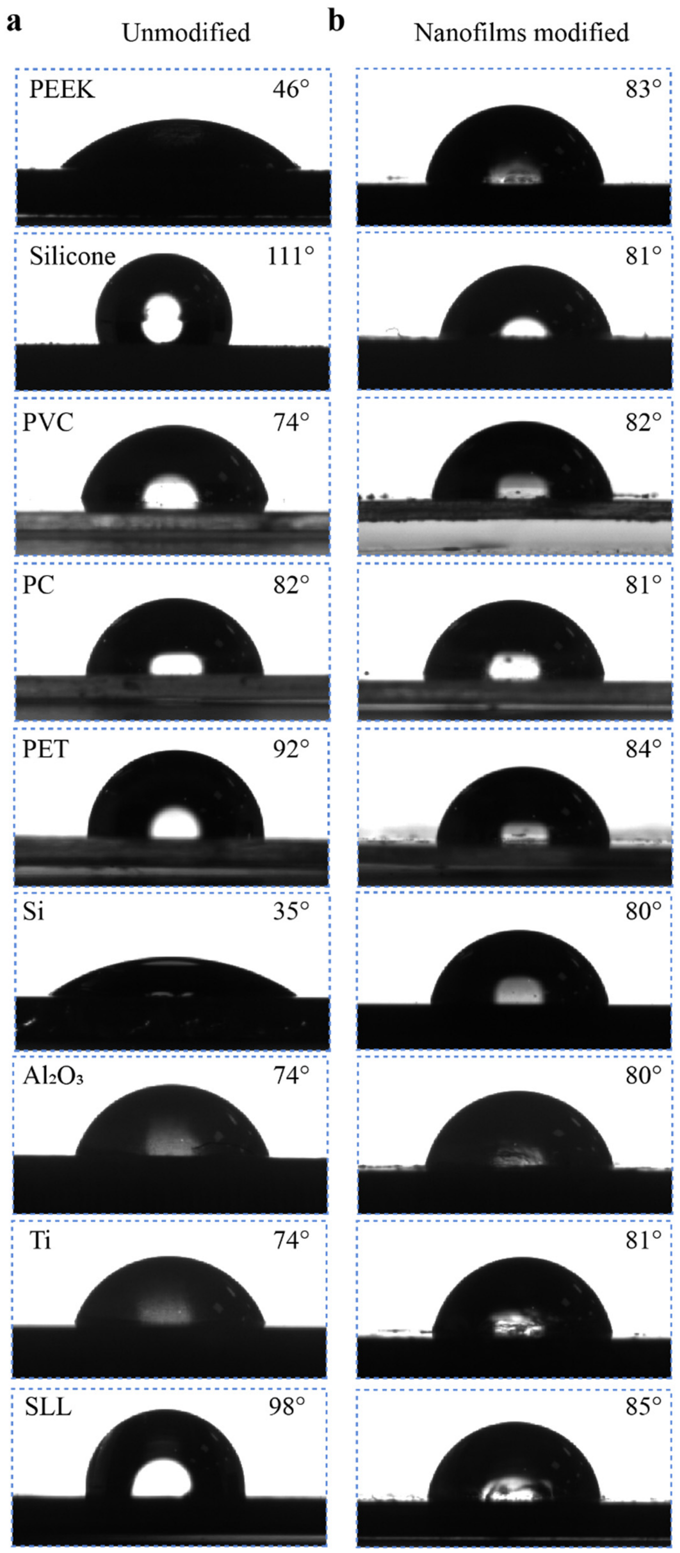

Supplementary Figure 7. The water contact angle images on unmodified substrates (a) and substrates coated by the hybrid PTB@SA nanofilm (b). The final concentrations of BSA and TCEP in the phase transition solution to prepare the coating were 8 mg/mL and 20 mM, respectively, and the mass ratio of SA to BSA was 0.1:1. PEEK: polyether ether ketone; PVC: polyvinyl chloride; PC: polycarbonate; PET: polyethylene terephthalate; Si: silicon wafer; Al_2O_3: aluminum oxide; Ti: titanium; SLL: stainless steel.

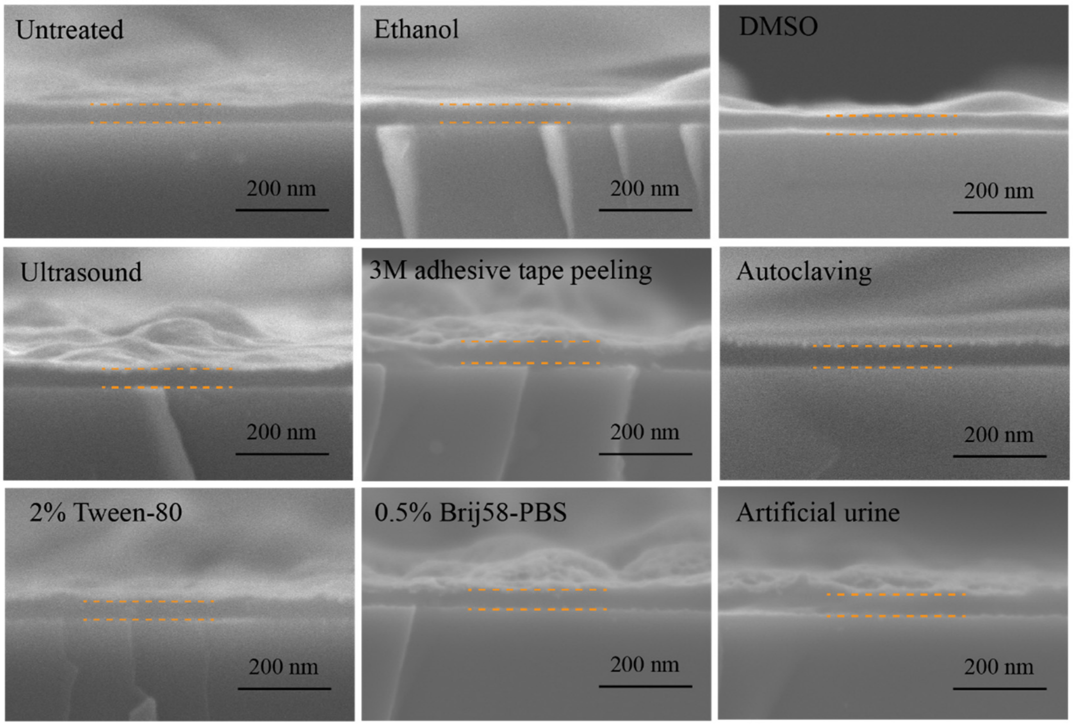

Supplementary Figure 8. The SEM cross-sectional images showing the thickness of the hybrid PTB@SA coating with an original thickness of 50 nm after different treatments. The dotted line indicates the coating. DMSO: dimethyl sulfoxide; Brij58: polyethylene glycol hexadecyl ether. The experiments were repeated independently at least three times with similar results.

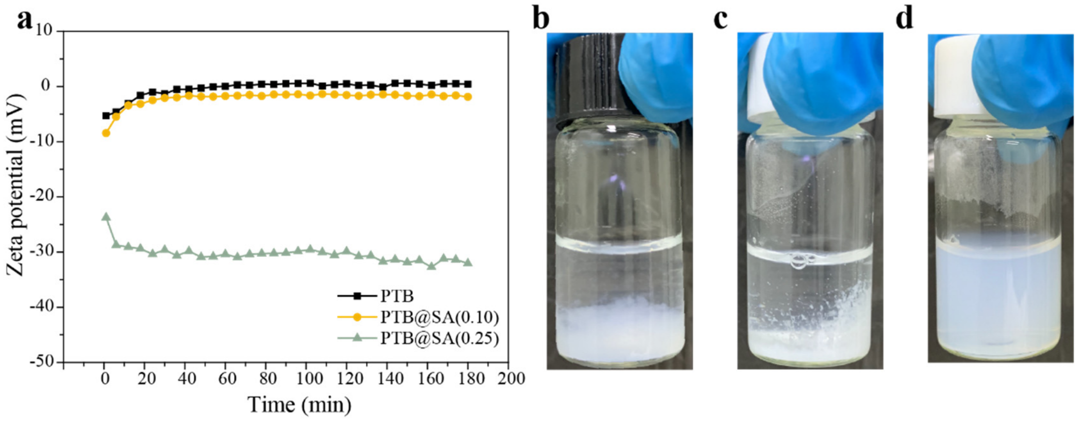

Supplementary Figure 9. Real-time zeta potential analysis of PTB, PTB@SA(0.10) and PTB@SA(0.25). (**a**) The zeta potential of the protein colloids in the phase transition solution with different addition ratios of SA incubated at 30°C for 3 h. (**b-d**) Corresponding optical images of the aggregation in the phase transition solution with different components. (**b**) PTB, (**c**) PTB@SA(0.10), (**d**) PTB@SA(0.25).

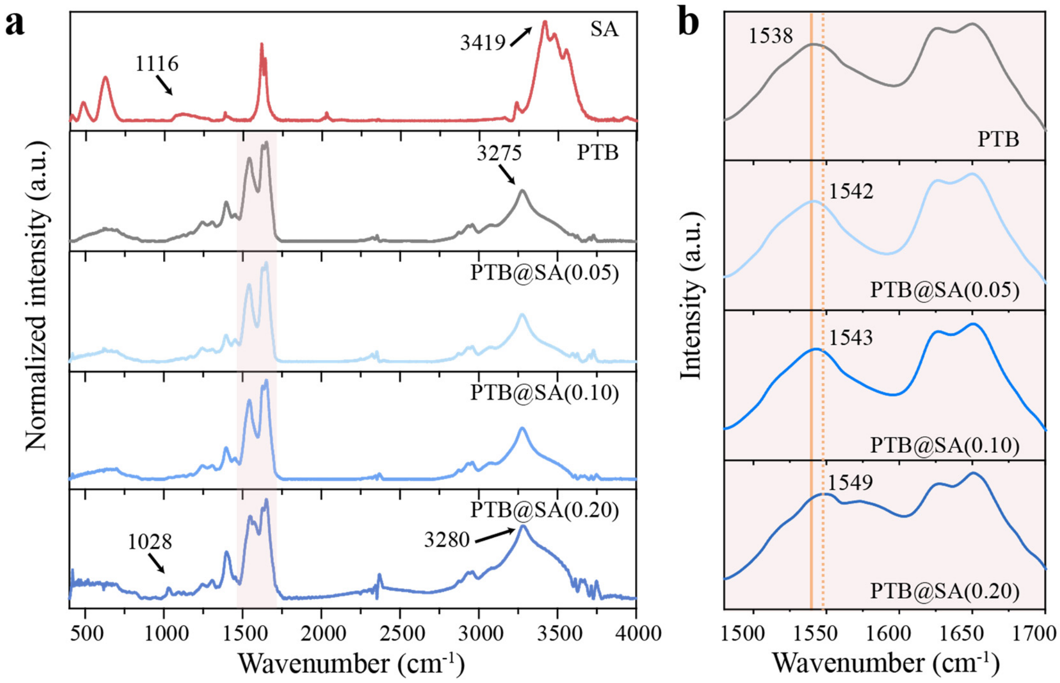

Supplementary Figure 10. FTIR characterization of SA, pure PTB nanofilm and PTB@SA nanofilm with different SA addition ratios. (**a**) FTIR spectra of the SA, PTB and hybrid PTB@SA nanofilms with different SA additive proportions. (**b**) The shift of amide II peak position with increasing the amount of SA integrated in the hybrid PTB@SA nanofilm.

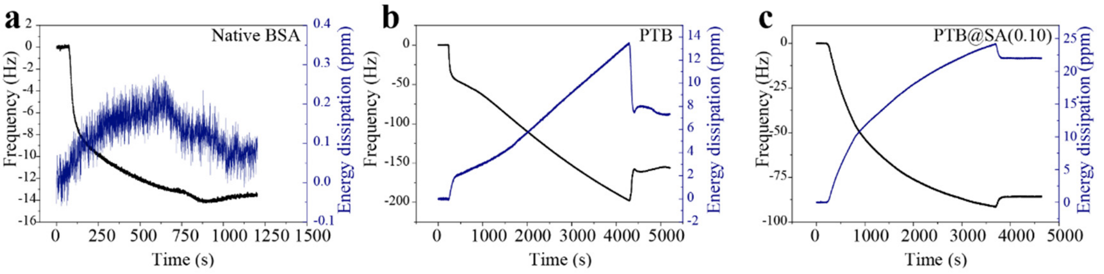

Supplementary Figure 11. QCM frequency shift and dissipation shift as a function of time during the adsorption of (a) native BSA, (b) phase transition solution of BSA (PTB), (c) phase transition solution of BSA doped with SA (PTB@SA) on Au surface. The concentration of native BSA was 0.33 mg/mL. After mixing 2 mL of BSA (2 mg/mL) with 2 mL of water, 2 mL of TCEP solution (50 mM, pH at 4.5) was added to the system to obtain a phase transition solution of BSA. The phase transition solution of BSA doped with SA was prepared by mixing 2 mL of BSA (2 mg/mL) with 1.2 mL of water and 0.8 mL of SA (1 mg/mL) solution, followed by the addition of 2 mL of TCEP (50 mM, pH at 4.5). Five minutes after the phase transition triggered by TCEP, the phase transition solution was filtered by a 0.22 μm filter and then pumped into the flow chamber. The final concentration of BSA in the above three solutions was 0.33 mg/mL.

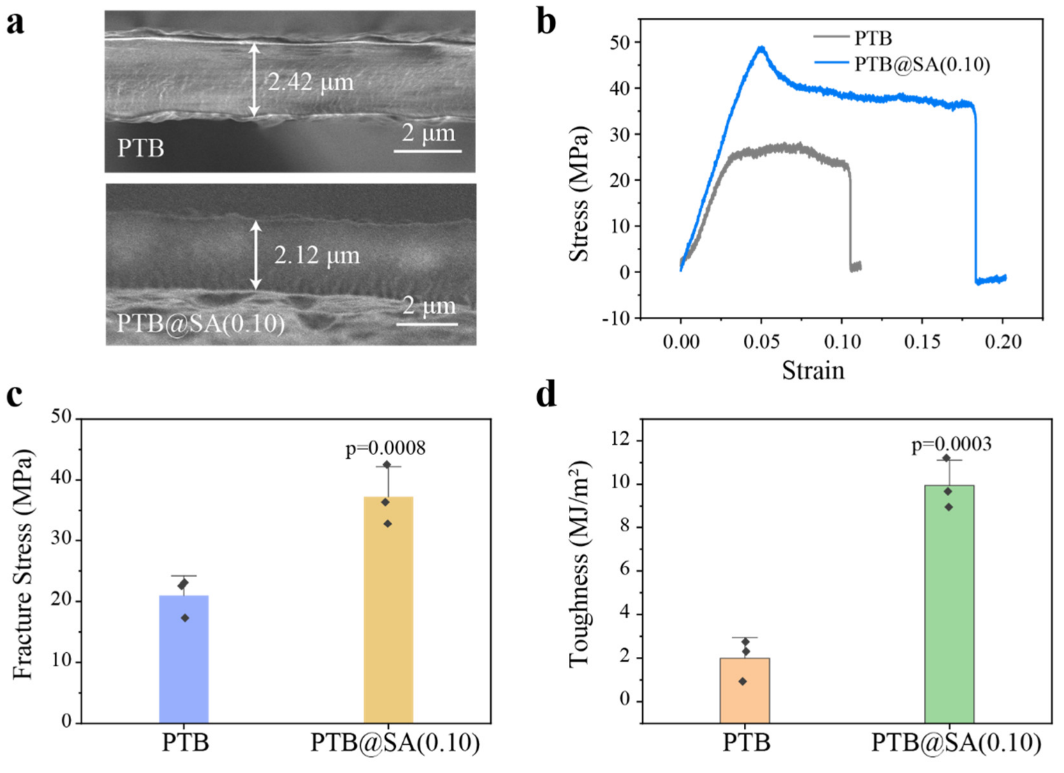

Supplementary Figure 12. The mechanical properties of the PTB and PTB@SA hybrid membrane measured by uniaxial tensile tests. (**a**) Cross-section images of the PTB or PTB@SA(0.10) films that were prepared by repeatedly stacking the nanofilms formed at the air/liquid interface. (**b-d**) Stress-strain curve (**b**), fracture stress (**c**), and toughness (**d**) of the PTB and PTB@SA(0.10) samples recorded at 22℃ and 75% relative humidity. Data are presented as the Mean ± S.D. n=3 independent samples per group. This figure was recorded at a higher ambient humidity than that of Fig. 3d, e. Increased humidity hydrated the proteinaceous films and thus reduced fracture stress with an accompanying increase in elongation at break. Statistical significance was determined by two-tailed Student's t-test.

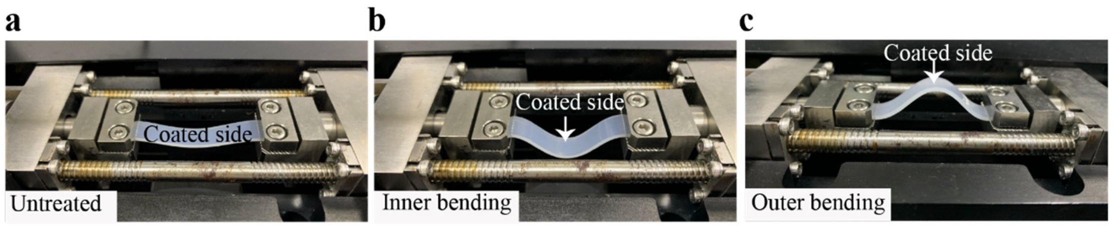

Supplementary Figure 13. The images showing each step of the bending test with a bending radius of 2 cm. (**a**) Untreated, (**b**) Inner bending, (**c**) Outer bending.

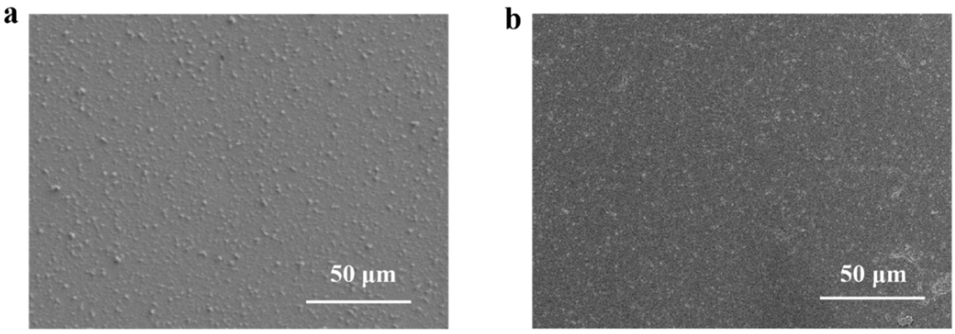

Supplementary Figure 14. SEM images of the PTB (a) or PTB@SA(0.10) (b) nanofilm-modified SR before the fatigue bending test. The experiments were repeated independently at least three times with similar results.

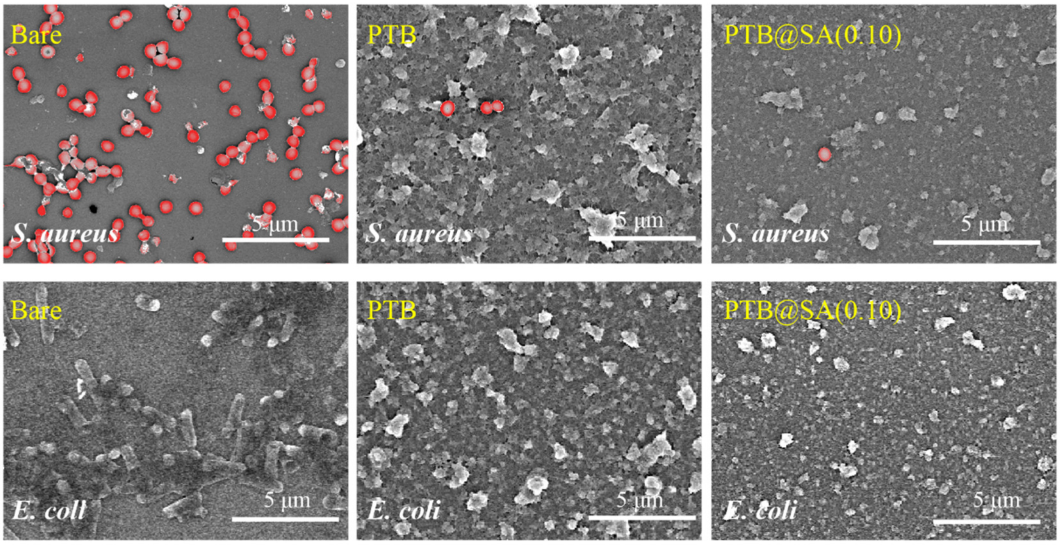

Supplementary Figure 15. Representative SEM images of *S. aureus* and *E. coli* adhered to bare substrate, PTB and PTB@SA(0.10) coating. The experiments were repeated independently at least three times with similar results.

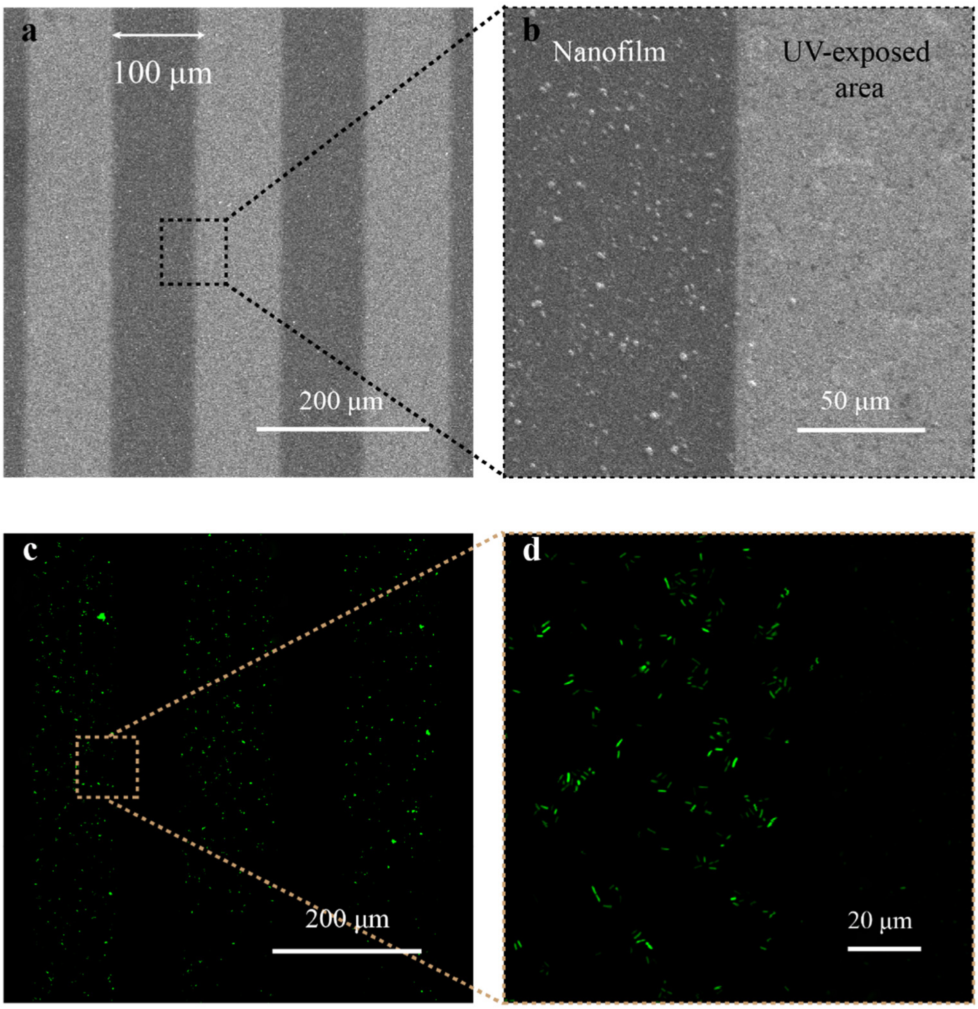

Supplementary Figure 16. (a, b) SEM images showing micropatterning of the PTB@SA(0.10) coating at the feature size of 100 µm. The experiments were repeated independently at least three times with similar results. **(c, d) CLSM images showing the adherence of *E. coli* on the bare substrate with micropatterned PTB@SA coating as the resistant layer.**

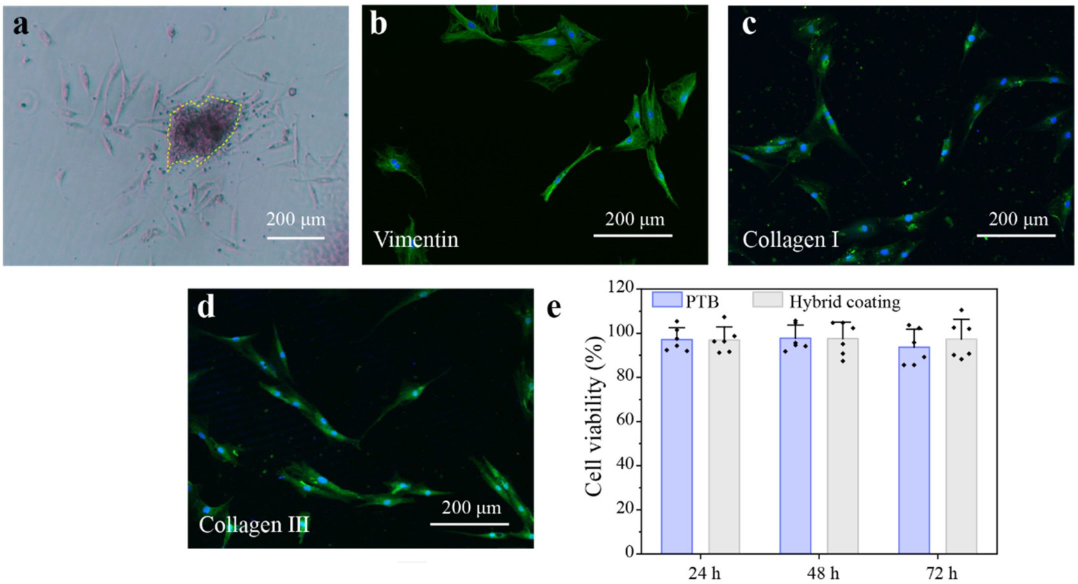

Supplementary Figure 17. Characterization and cytotoxicity evaluation of primary urethral fibroblasts established from fresh rat urethral tissue. (**a**) The fibroblasts isolated from urethral tissues presented typical spindle shape under an optical microscope. The dotted line showing the fragments of urethral tissue after collagenase digestion. (**b-d**) The expression of typical fibroblast protein markers in the primary fibroblast as determined by immunofluorescences staining. The fibroblasts were stained positive for Vimentin (**b**), Collagen I (**c**), and Collagen III (**d**) under a fluorescent microscopy. The nuclei were counter-stained with DAPI. (**e**) The cytotoxicity of PTB and PTB@SA(0.10) coating on rat primary urethral fibroblasts analyzed by MTT assay. Data are presented as the Mean ± S.D. n=6 independent cells per group.

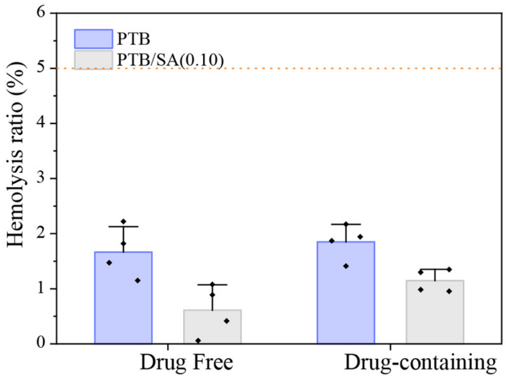

Supplementary Figure 18. The hemolysis ratios of the PTB, PTB@SA(0.10) nanofilms and the corresponding rapamycin-loaded coatings. All values were below 5%. Data are presented as the Mean ± S.D. n=4 independent samples per group.

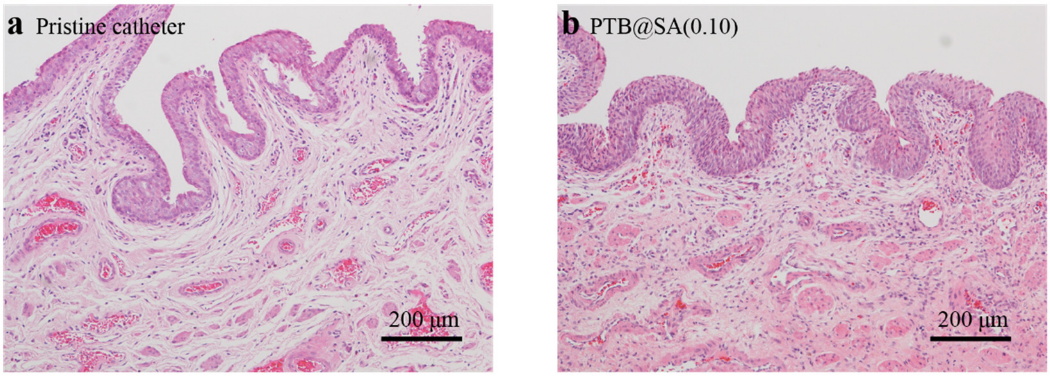

Supplementary Figure 19. H&E staining of rabbit urethral tissue after indwelling the pristine SR catheter (a) or PTB@SA(0.10) coated urinary catheter (b) for 1 month to evaluate histocompatibility. n=5 animals per group.

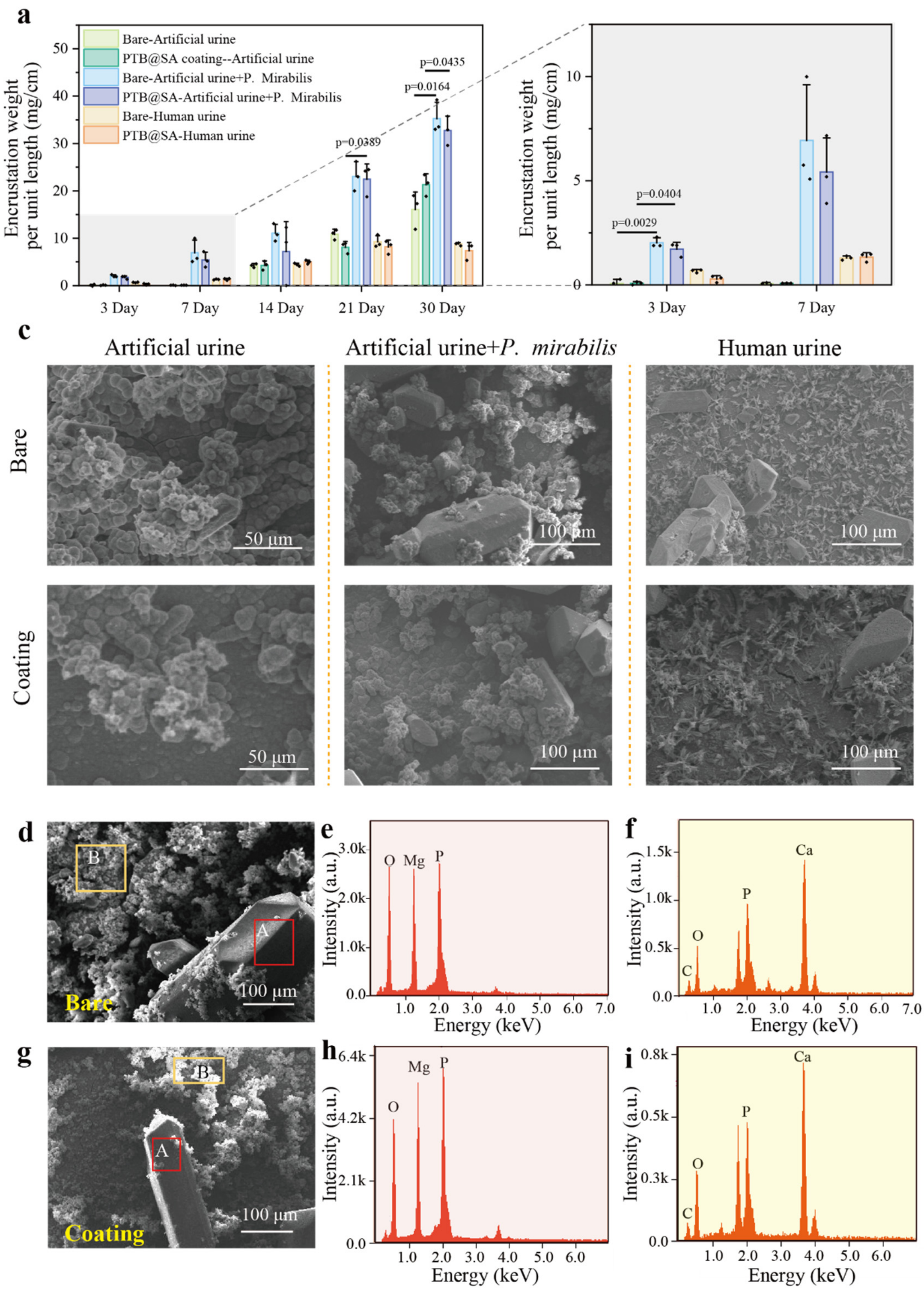

Supplementary Figure 20. In vitro evaluation of the encrustation performance of bare silicone catheters and the PTB@SA nanofilm-modified catheters. (**a**) The weight of crystals deposited on bare catheters or the PTB@SA coated catheters after exposure to artificial urine, artificial urine containing *Proteus mirabilis* (*P. mirabilis*), or human urine. Data are presented as the Mean ± S.D. n=3 independent samples per group. (**b**) The weight of crystals deposited on bare catheters or the PTB@SA coated catheters during the first week. Data are presented as the Mean ± S.D. n=3 independent samples per group. (**c**) SEM of bare catheter and the PTB@SA coating after 1 month

of encrustation experiment in vitro. (**d**) SEM image of bare catheter surface after encrustation experiment. (**e, f**) EDS analysis of area A (**e**) and B (**f**) in **Supplementary Figure 20 d**. (**g**) SEM image of the PTB@SA coating surface after encrustation experiment. (**h, i**) EDS analysis of area A (**h**) and B (**i**) in **Supplementary Figure 20 g**. Statistical significance was determined by two-way ANOVA with Tukey's multiple comparisons test (**a**, **b**). More statistical information is presented in the source data.

Supplementary method:

Sterile sample cups were used as the reaction vessels, which contained 120 mL of artificial urine, artificial urine suspended with *P. mirabilis* (10^5 CFU/mL), and human urine, respectively. The bare or PTB@SA nanofilm-modified urinary catheters segments (each with a length of 1 cm) were suspended in urine through sterile needles fixed on the container lids. The above vessels were placed in an orbital shaker so that all samples were at a consistent physiological temperature of 37°C and a rotational speed of 70 r/min. Every day, 60 mL of urine in the container was replaced by fresh corresponding urine in a standard laboratory biosafety cabinet. At the indicated time points, samples were removed and rinsed three times with ultrapure water to wash away dissolved salts. All samples were individually weighed after drying and compared with their initial weight to calculate the weight of the encrustation. Next, the morphology and elemental composition of the surface crusts were analyzed using SEM and Energy Dispersive Spectroscopy (EDS).

Supplementary discussion:

Encrustation is a thorny problem for patients who require long-term indwelling catheters, which involves the deposition of inorganic salts on the surface of the device or the inner wall of the lumen, and further leads to pain, difficult removal of catheter, blockage of urinary flow and intractable bacterial biofilms. Two important aspects that promote the development of crusts include supersaturation of crystals in the urine and bacterial infection. To comprehensively compare the encrustation performance of bare catheters and PTB@SA-modified catheters, we performed a well-established in vitro experiment in artificial urine, artificial urine in the presence of *P. mirabilis* or human urine, respectively[2]. As shown in Supplementary Fig.20 a and b, there was no difference in encrustation weight between the control and PTB@SA-coated samples exposed to the same urine environment at each time point, except that the weight of crystals formed in human urine on day 3 was smaller in the PTB@SA group. At the same time point, the presence of *P. mirabilis* significantly aggravated the encrustation, because the urease produced by *P. mirabilis* would increase the pH of urine and promote the deposition of calcium phosphate and magnesium. After 1 month, both the bare catheter and the PTB@SA coated surfaces were covered with cauliflower-like smooth deposits and interspersed with large coffin-like crystals (Supplementary Fig.20 c). Furthermore, we performed Energy Dispersive Spectroscopy (EDS) analysis of these deposits and determined that the smooth layer was mainly composed of Ca, C, P and O, presumably being calcium carbonate apatite crystallites. while the large coffin-shaped crystals were composed of Mg, O and P, which could be assigned to struvite (magnesium phosphate) (Supplementary Fig.20 d-i). Overall, the PTB@SA nanofilm did not induce more severe encrustation formation on the device surface within 30 days, indicating that it would not pose an additional threat when applied to construct functional urinary catheters.

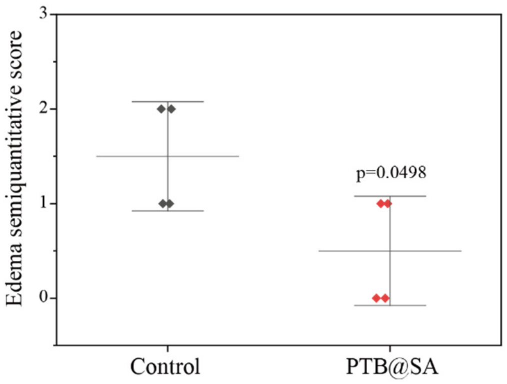

Supplementary Figure 21. Quantitative assessment of the edema severity in control and PTB@SA-coated groups. Data are presented as the Mean ± S.D. n=4 independent samples per group. Statistical significance was determined by two-tailed Student's t-test.

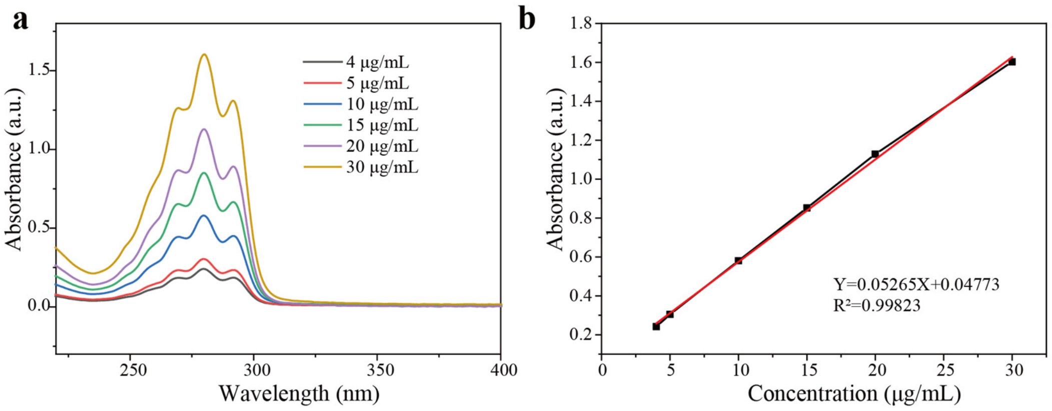

Supplementary Figure 22. Establishment of regression equation for rapamycin dissolved in release medium. (**a**) UV-Vis spectra of rapamycin dissolved in 0.5% Brij58-PBS solution at different concentrations. (**b**) The standard curve and regression equation of rapamycin in the release medium.

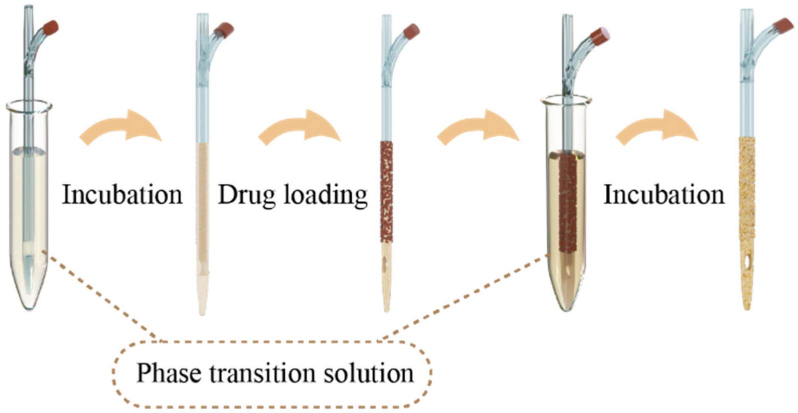

Supplementary Figure 23. Schematic diagram for the preparation of the anti-fibrotic drug coating on the urethral catheter for continuous local drug delivery. This drug delivery system consists of a sandwich-like structure including the bottom priming layer of PTB@SA(0.10), the middle solid drug reservoir deposition layer and the top release rate-controlling membrane of PTB@SA nanofilm.

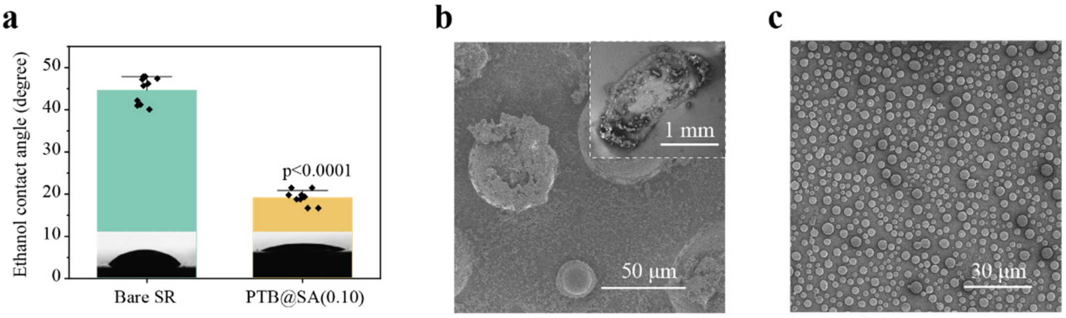

Supplementary Figure 24. Improved effect of the PTB@SA(0.10) priming coating on poor wettability of SR and uniform distribution of drug particles. (**a**) The contact angle of ethanol on the surface of bare SR and the PTB@SA(0.10) coating. These values indicate that the hybrid coating could improve the wettability of ethanol on the surface of SR. Data are presented the Mean ± S.D. n=10 independent samples per group. Statistical significance was determined by two-tailed Student's t-test. (**b**) The SEM images of drug gathered on bare SR, with the inset showing an image of 500 times magnification. (**c**) The SEM image of solid drug particles uniformly dispersed on the SR modified with PTB@SA(0.10) nanofilm. The presence of the hybrid PTB@SA nanofilm priming layer on SR surface allowed the drug-ethanol solution to spread evenly on hydrophobic SR surface. In contrast, due to the poor wettability of SR, the drug-containing ethanol cannot be uniformly dispersed on the SR without the PTB@SA priming layer, and the resultant drug particles easily aggregated together.

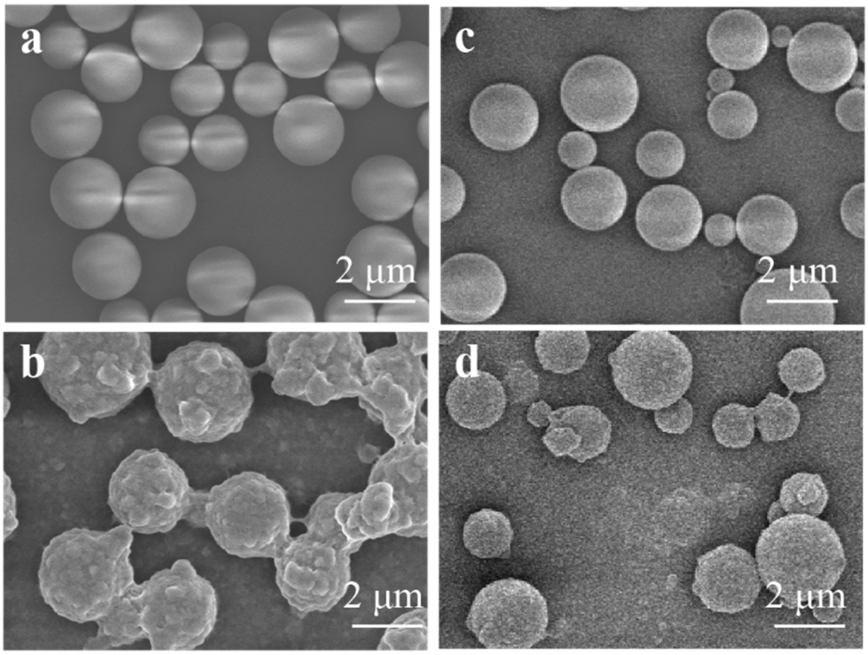

Supplementary Figure 25. The SEM images showing the bare rapamycin particles on the PTB (a) or PTB@SA(0.10) (c) priming coating, and the morphology of the drug particles coated with the PTB (b) or PTB@SA(0.10) nanofilm (d). Before loading the drug, the surface of the wafer was modified with the corresponding PTB or hybrid PTB@SA priming coating. As the biopolymer nanoparticles assembled in situ on the reservoir to form a nanofilm coating, the originally smooth drug particles surface became rough.

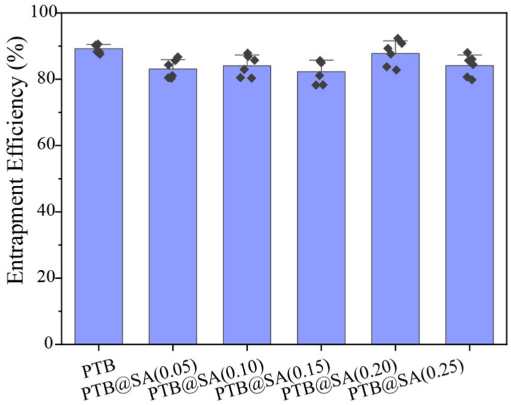

Supplementary Figure 26. Drug entrapment efficiency of the delivery systems with the release-rate controlling PTB@SA nanofilms at different content of SA. Data are presented as the Mean ± S.D. n=6 independent samples per group.

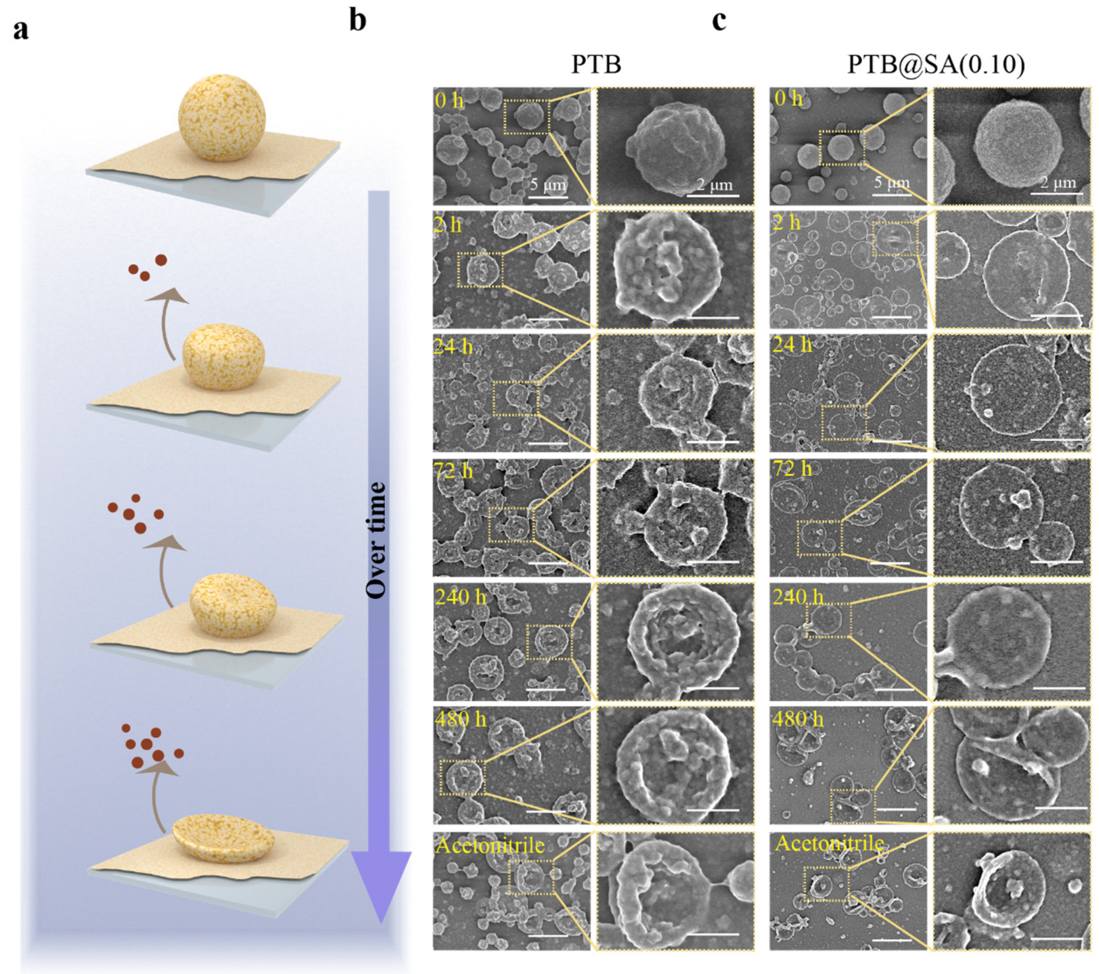

Supplementary Figure 27. The morphology of the drug delivery system tracked by SEM as a function of the release process. (**a**) The schematic diagram showing that the morphology of the drug reservoir coated by the PTB or PTB@SA(0.10) nanofilms changed with the drug release process. (**b**, **c**) The SEM images showing the morphology of the rapamycin delivery system controlled by the PTB (**b**) or PTB@SA(0.10) nanofilm (**c**) over time, as well as the morphology after the drug in the reservoir was completely eluted by acetonitrile. Scale bar on the left is 5 μm, and scale bar on the right is 2 μm. The occurrence and progress of the collapse of solid drug reservoir could be reflected by the area and extent of the central depression of the reservoir. Therefore, as the drug release progressing, the rapamycin spheres coated with PTB or PTB@SA(0.10) nanofilm gradually collapsed and approached the state of elution with acetonitrile, indicating a long-term release.

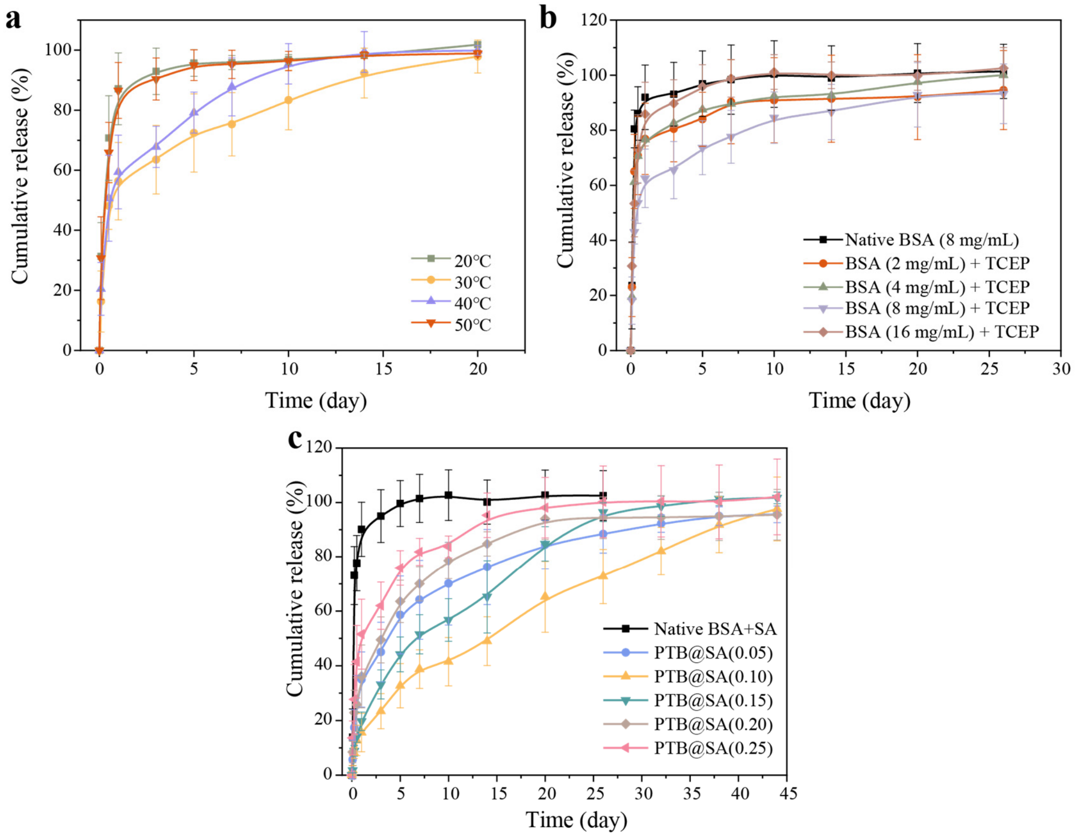

Supplementary Figure 28. Rapamycin release behavior controlled by different release-rate controlling membranes. (**a**) The release behaviors of rapamycin controlled by the PTB nanofilm prepared at different temperatures. (**b**) The release profiles of rapamycin as a function of the final concentration of BSA used to prepare the release-rate controlling PTB membrane, with the use of the non-specific adsorption layer of native BSA as a control. (**c**) The release profile of rapamycin as a function of the mass ratio of SA to BSA (from 0.05:1 to 0.25:1), with the final concentration of BSA being kept at 8 mg/mL and the non-specific adsorption layer of the mixture of native BSA and SA was used as a control. Data are presented as the Mean ± S.D. n=6 independent samples per group.

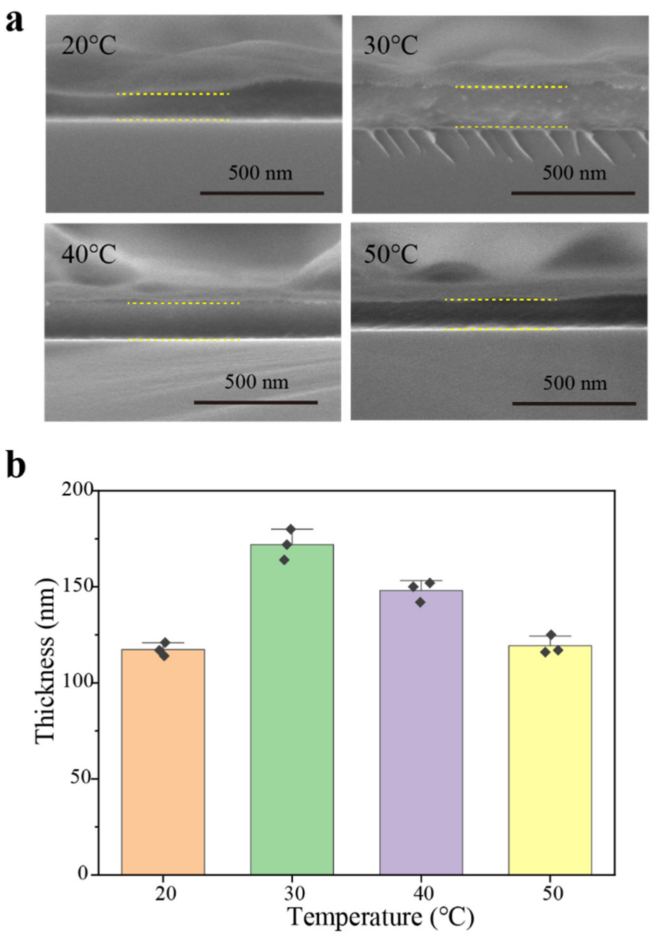

Supplementary Figure 29. The effect of incubation temperature on the thickness of the PTB nanofilm. (**a**) The SEM cross-sectional images showing the thickness of the PTB coating prepared at different temperatures. The dotted line indicates the coating. (**b**) The thickness of the PTB coating controlled by the reaction temperature. The silicon wafer was immersed in the phase transition solution obtained by mixing BSA (20 mg/mL), water and TCEP (50 mM, pH=4.5) in a volume ratio of 1:1:0.5, and incubated at the indicated temperature for 180 min. Data are presented as the Mean ± S.D. n=3 independent samples per group.

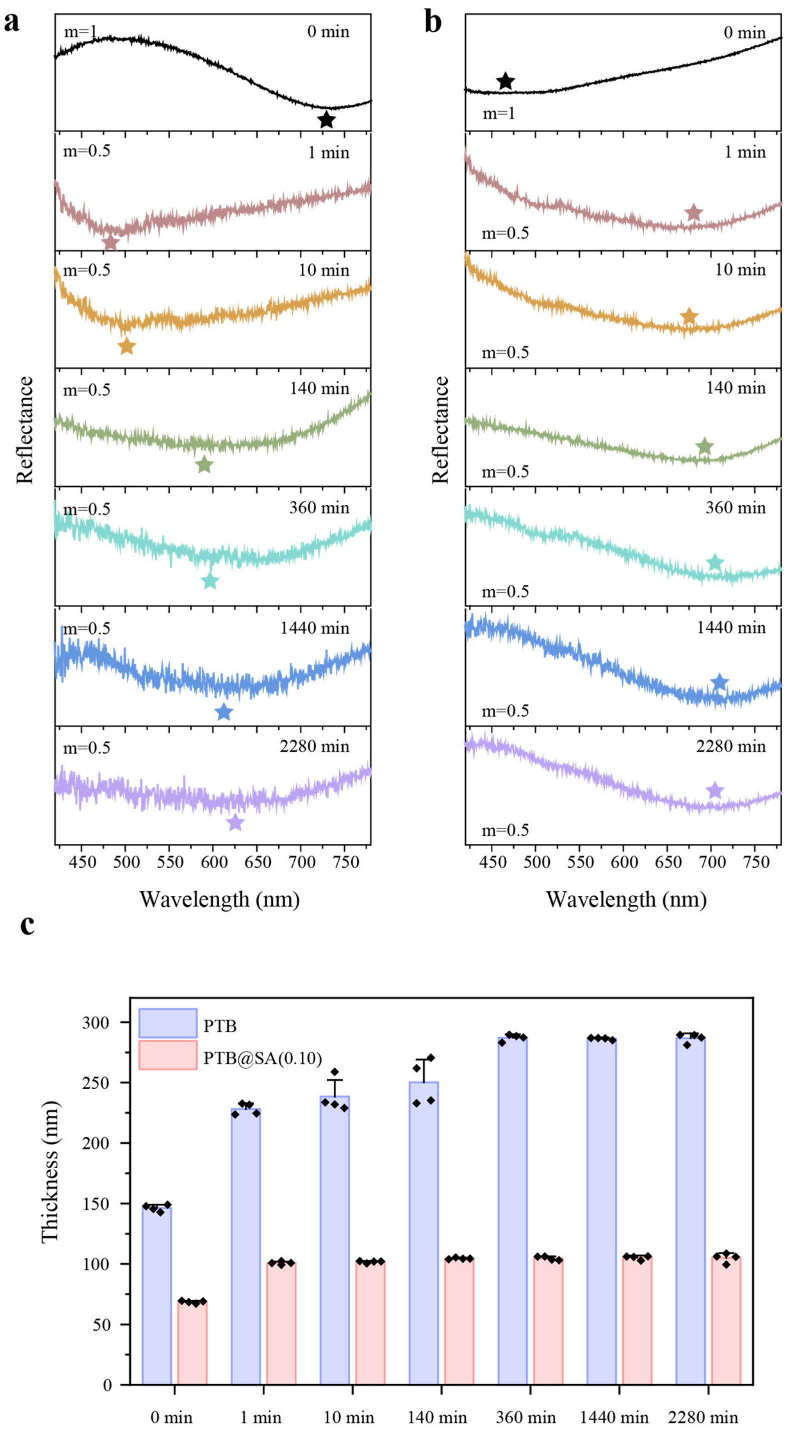

Supplementary Figure 30. The thickness of the PTB and PTB@SA nanofilms as a function of immersion time in the release medium. The corresponding reflection spectra of the PTB (**a**) and PTB@SA(0.10) nanofilm (**b**) at an incident angle of 0° with different immersion time in the release medium. Asterisks indicate reflection valleys. (**c**) The thickness variation of the PTB and PTB@SA(0.10) nanofilm in the release medium as the function of time. Data are presented as the Mean ± S.D. n=4 independent samples per group.

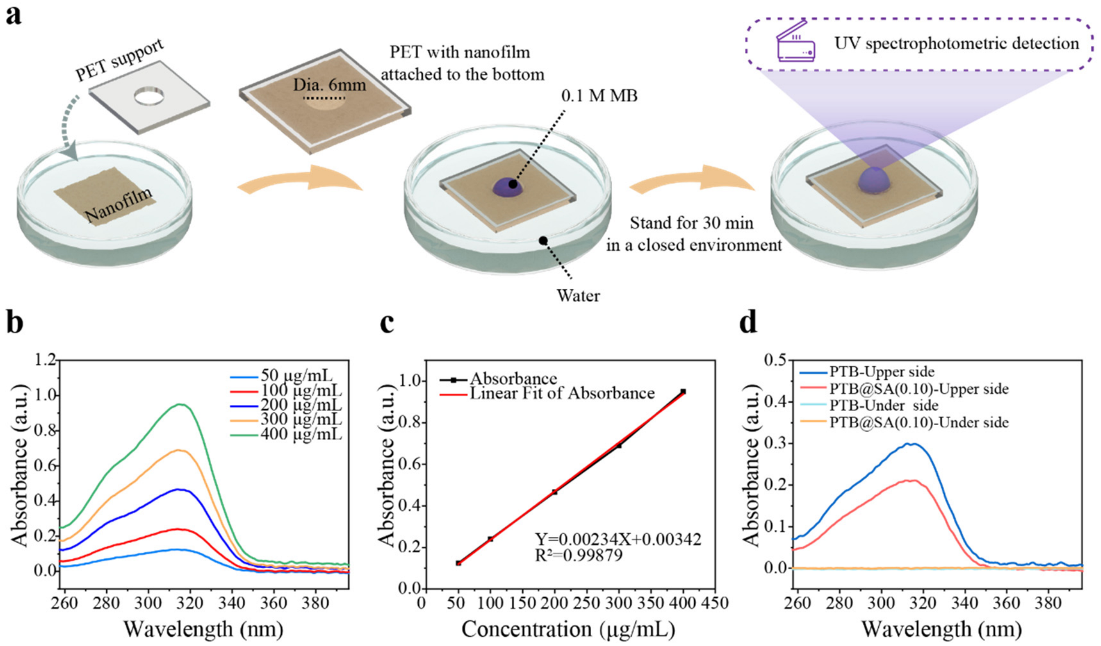

Supplementary Figure 31. The water permeability of the PTB or PTB@SA nanofilm was determined by forward osmosis. (**a**) Schematic diagram of water permeability of the PTB or PTB@SA(0.10) nanofilm determined by forward osmosis. Methyl blue (MB, 0.1 M), which could be completely retained by the nanofilm, was applied to the upper side (air side) of the nanofilm to provide a constant driving force for permeation. (**b**) UV-Vis spectra of MB aqueous solutions at different concentrations. (**c**) Standard curve and regression equation of MB aqueous solution. (**d**) After 30 min, the MB solution located on the upper side of the PTB or PTB@SA(0.10) nanofilm in **(a)** (PTB-Upper side or PTB@SA Upper side) was diluted by the water permeating through the nanofilm, and the resulting decrease in MB concentration was used to estimate the water flux and permeability of the nanofilm. In addition, there is no absorption in the UV spectrum of the water under the nanofilm (PTB-Under side or PTB@SA Under side), indicating that the rejection ratio of MB for both PTB and PTB@SA(0.10) nanofilm was 100%.

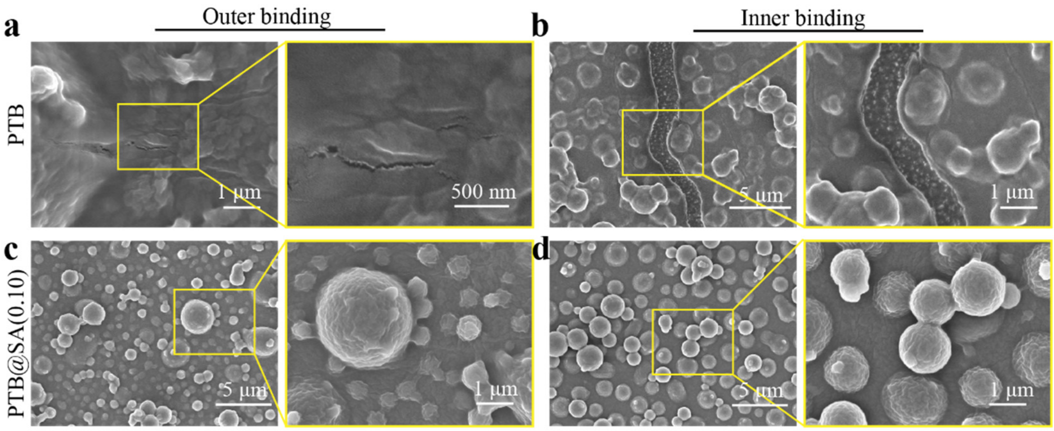

Supplementary Figure 32. The SEM images showing the integrity of the rapamycin delivery system controlled by the PTB or PTB@SA(0.10) nanofilm on the SR after the bending test. (**a**) The delivery system using the PTB nanofilm as the release-rate controlling membrane after suffering outer bending with a bending radius of 1.5 cm. (**b**) The delivery system using the PTB nanofilm as the release-rate controlling membrane after suffering inner bending with a bending radius of 1.5 cm. (**c**) The delivery system using the PTB@SA(0.10) nanofilm as the release-rate controlling membrane after suffering outer bending with a bending radius of 1.5 cm. (**d**) The delivery system using the PTB@SA(0.10) nanofilm as the release-rate controlling membrane after suffering inner bending with a bending radius of 1.5 cm.

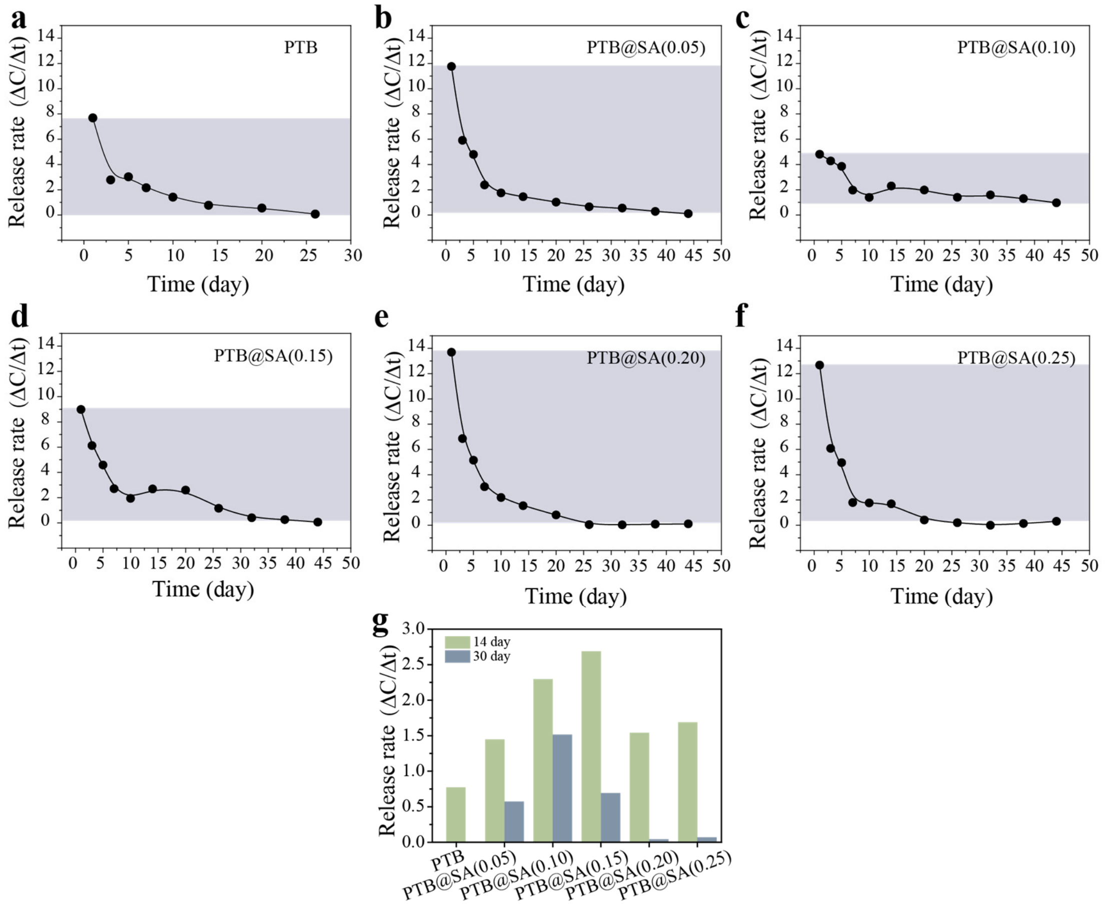

Supplementary Figure 33. Effect of SA additive proportion in phase transition solution used to prepare release-rate controlling membrane on rapamycin release rate. (**a-f**) The effect of the addition ratio of SA in the phase transition solution on the release rate of rapamycin, with using the PTB or PTB@SA at different SA addition ratio as the release-rate controlling membrane. The release rate was derived from the derivative of the cumulative release, meaning the average percentage of the drug released per day at the indicated time. Among them, PTB@SA(0.10) exhibited the most stable release rate throughout the release period. (**g**) The release rate of rapamycin controlled by the nanofilms with different SA additive proportions on the 14th and 30th day. The initial moderate release rate contributed to the reservation of sufficient dose for the later period. Therefore, at the 30th day, rapamycin was still being released from the PTB@SA(0.10) nanofilm at a rate of 1.52% of the total drug amount per day, which was much higher than that of membranes prepared in other proportions.

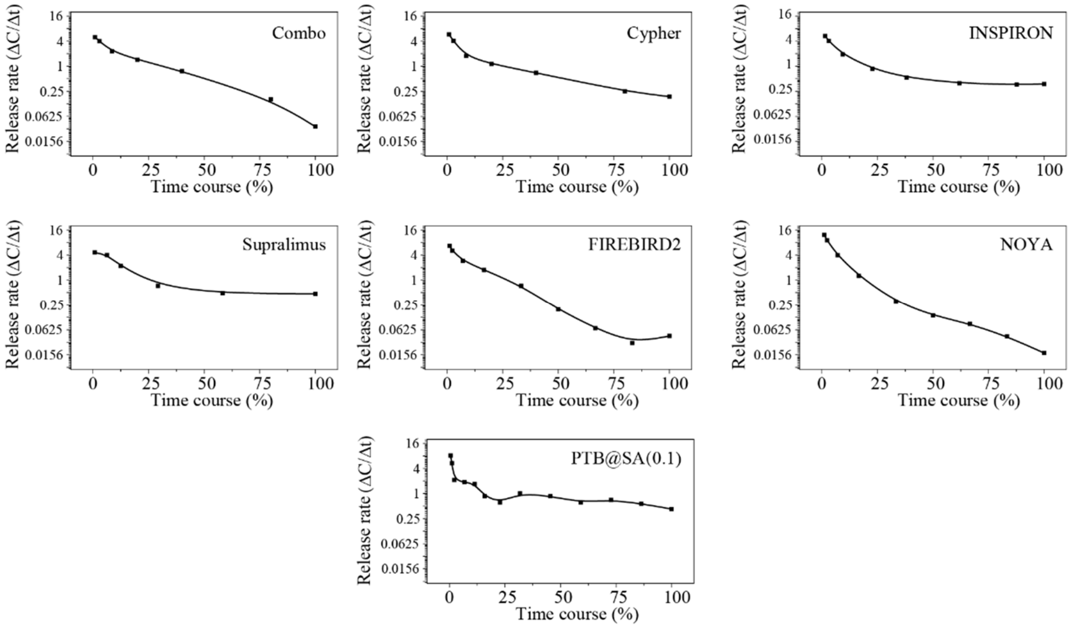

Supplementary Figure 34. Rapamycin release rate controlled by the PTB@SA(0.10) nanofilm and commercially available rapamycin-eluting stents, including Combo, Cypher, INSPIRON, Supralimus, FIREBIRD2 and NOYA[3–6]. The detailed information of commercially available products is listed in Supplementary Table 3.

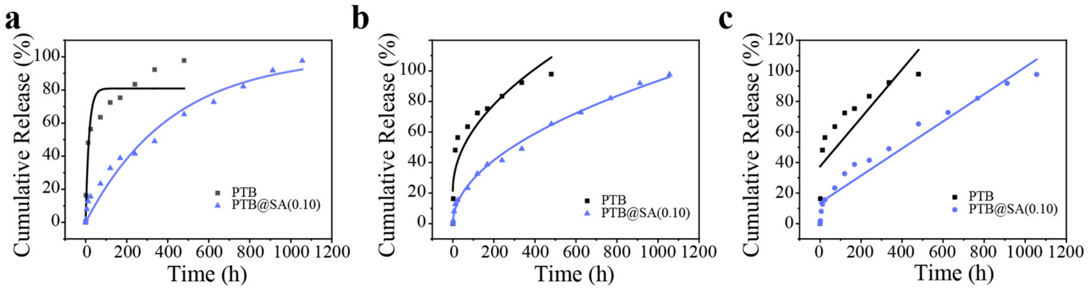

Supplementary Figure 35. Mathematical models for drug release kinetics by using the PTB or PTB@SA(0.10) nanofilm as the release-rate controlling membrane. (**a**) First-order model, (**b**) Higuchi model, (**c**) Zero-order model. The correlation coefficient (R^2) and regression equations for each model are presented in the Supplementary Table 4.

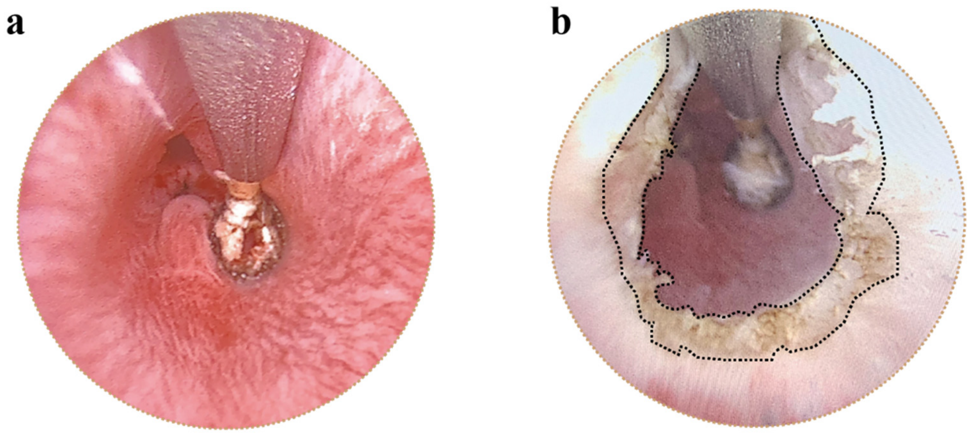

Supplementary Figure 36. Representative urethroscopy before (a) and after (b) urethral electrocoagulation in rabbits. The dotted line indicates the boundary between the urethra damaged by electrocoagulation and the adjacent normal tissues.

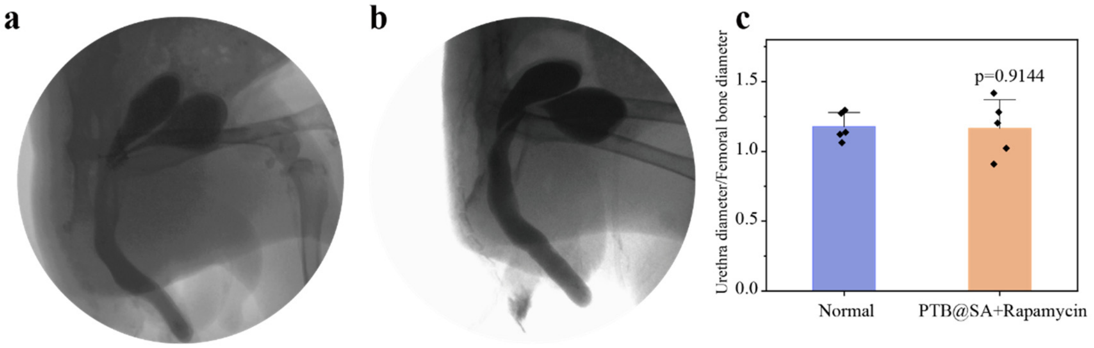

Supplementary Figure 37. Comparison of urethral diameter in normal healthy rabbits and rabbits using PTB@SA-Rapamycin catheter after urethral injury. (**a**) Representative retrograde urethrogram of healthy rabbits. (**b**) Representative retrograde urethrogram of the rabbit in the anti-fibrotic catheter group. (**c**) The ratio of urethra diameter to femoral shaft diameter in normal healthy rabbits and rabbits in anti-fibrotic catheter (PTB@SA-Rapamycin) group. Data are presented as the Mean ± S.D. n=5 animals per group. Statistical significance was determined by two-tailed Student's t-test.

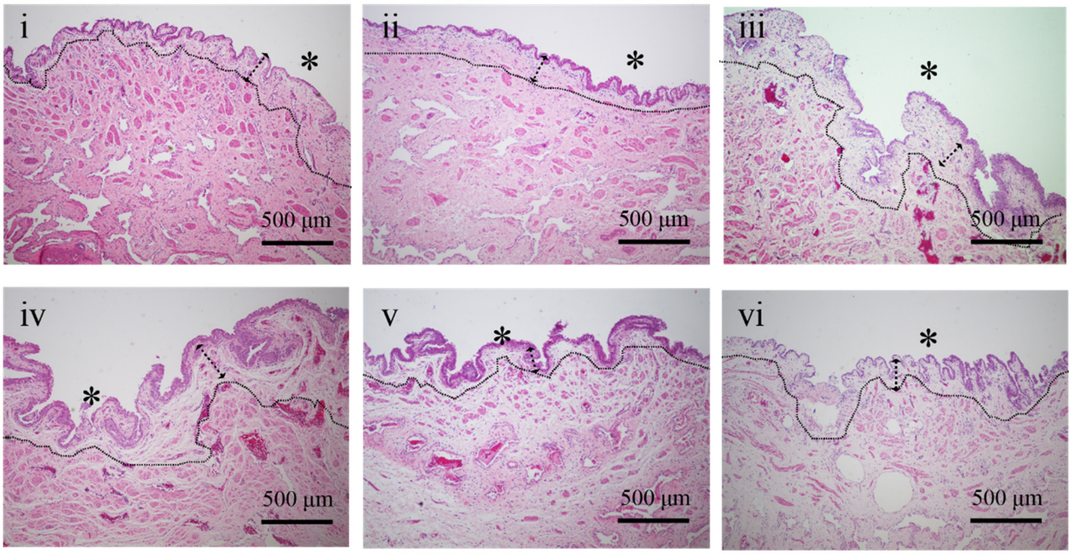

Supplementary Figure 38. H&E staining images of undamaged urethra after 1 month in each group. i, Control, ii; Unmodified catheter; iii, Systemic administration; iv, PTB@SA; v, Burst-releasing; vi, PTB@SA-Rapamycin. Asterisk represents urethral luminal side, and the dotted line indicates the boundary between the submucosa and the muscularis. n=5 animals per group.

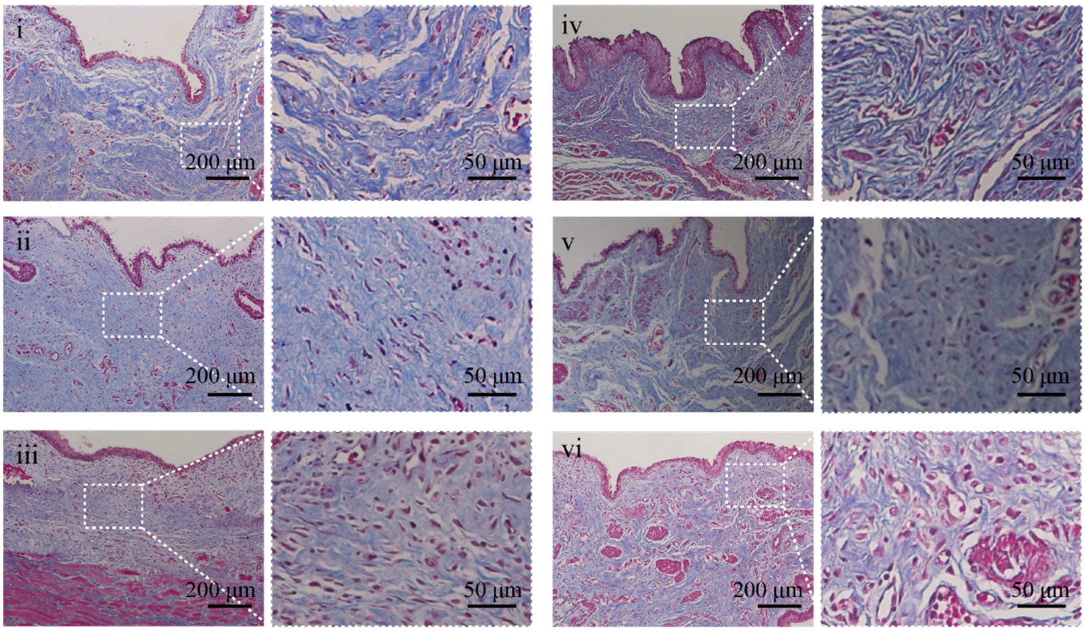

Supplementary Figure 39. Masson's Trichrome staining images showing collagen fiber deposition under the damaged urethral mucosa after being treated differently for one month. i, Control, ii; Unmodified catheter; iii, Systemic administration; iv, PTB@SA; v, Burst-releasing; vi, PTB@SA-Rapamycin. n=5 animals per group.

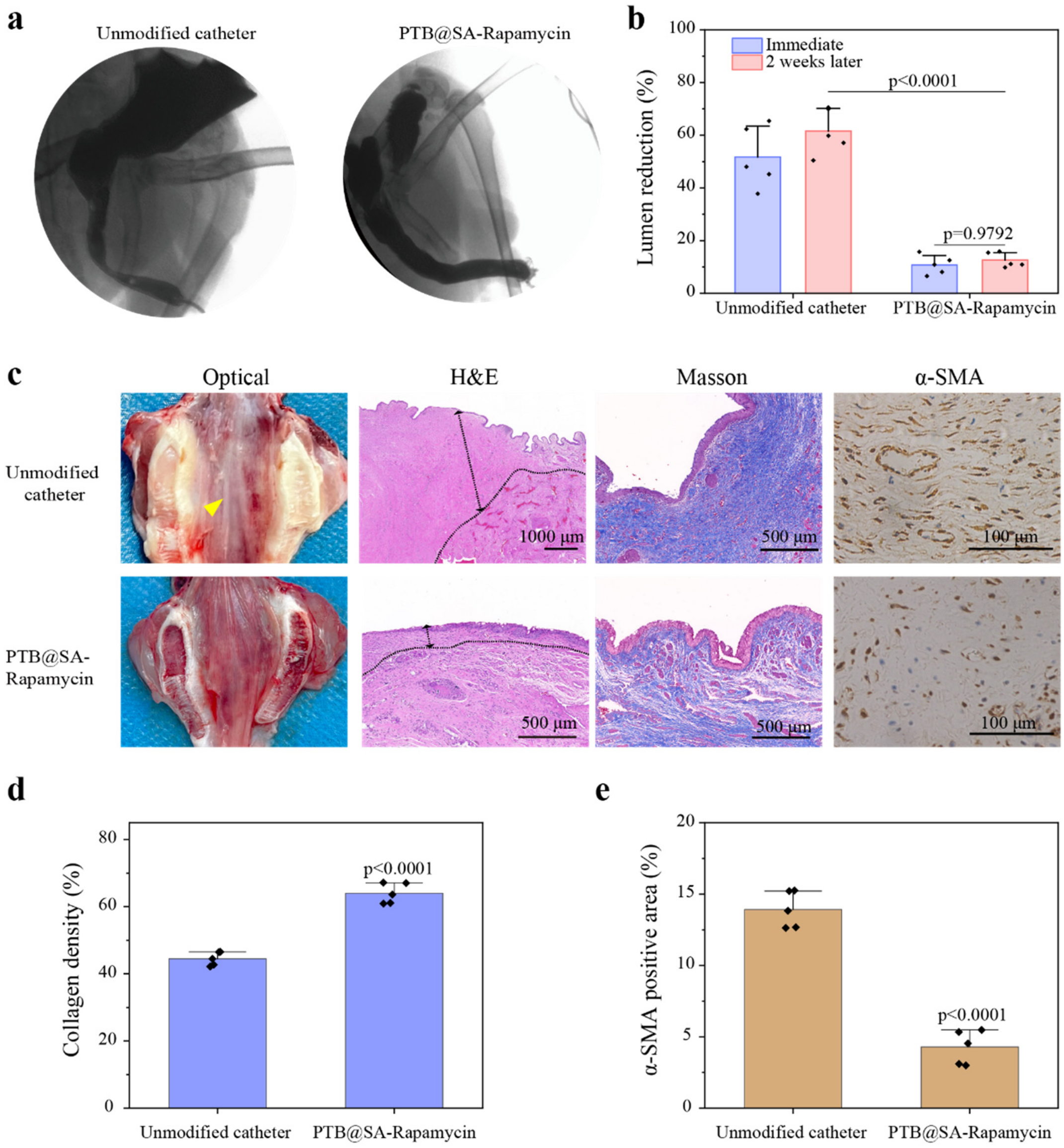

Supplementary Figure 40. Rabbit urethral stricture in unmodified urinary catheter group and PTB@SA-Rapamycin group on the 14th day after removal of catheter. (**a**) Representative retrograde urethrogram using 76% meglumine diatrizoate as contrast agent. (**b**) Lumen reduction immediately after catheter removal or 2 weeks later as determined by retrograde urethrography. n=5 animals per group . (**c**) Comparison of gross specimens, H&E staining, Masson staining and α-SMA expression between unmodified urinary catheter group and PTB@SA-Rapamycin group at 14 days after catheter removal. Yellow triangles indicate urethral scar tissue, and the dotted lines indicate the boundary between the submucosa and the muscularis. (**d, e**) Quantitative results of collagen density and α-SMA positive area in **Supplementary Figure 40c.** n=5 animals per group. All data are presented as the Mean ± S.D. Statistical significance was determined by two-way ANOVA with Tukey's multiple comparisons test (**b**) or two-tailed Student's t-test (**d, e**).

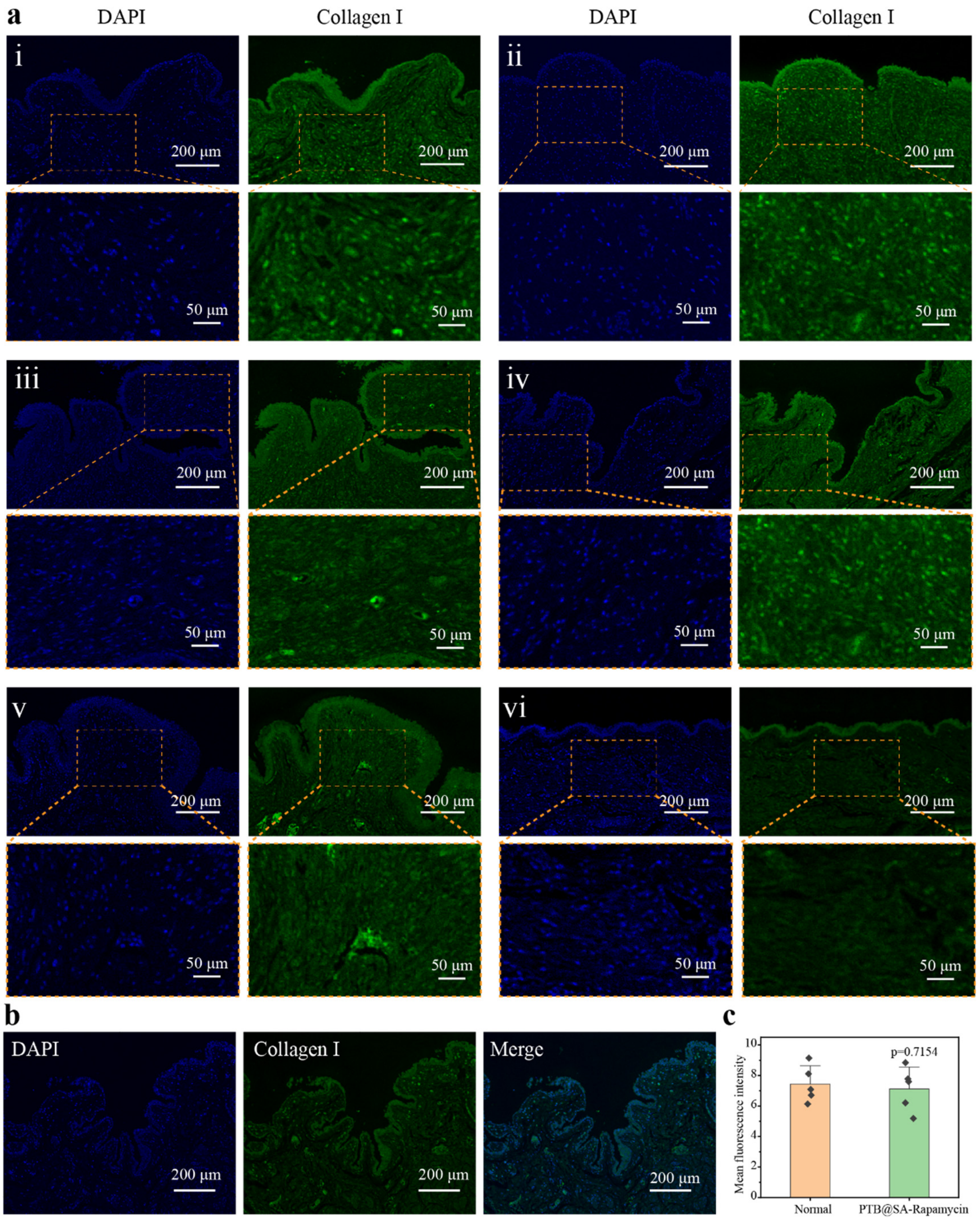

Supplementary Figure 41. Immunofluorescence staining images showing collagen deposition in urethral tissue. (**a**) Immunofluorescence staining of Collagen Ⅰ at the urethral injury site after different treatments for 1 month. Nuclei (blue) was stained with DAPI. i, Control, ii; Unmodified catheter; iii, Systemic administration; iv, PTB@SA; v, Burst-releasing; vi, PTB@SA-Rapamycin. (**b**) The urethral tissue of normal rabbits was sectioned for immunostaining with Collagen Ⅰ. Nuclei (blue) was stained with DAPI. (**c**) Quantification of the Collagen Ⅰ fluorescence intensity in normal rabbit urethral tissue and PTB@SA-Rapamycin catheter-mediated urethral injury healing. Data are presented as the Mean ± S.D. n=5 animals per group. Statistical significance was determined by two-tailed Student's t-test.

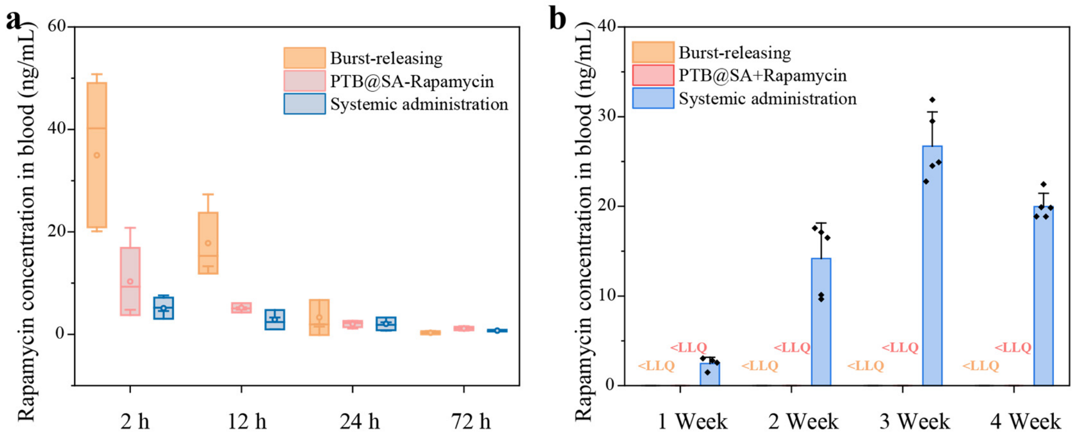

Supplementary Figure 42. Circulatory distribution of rapamycin in systemic administration, burst-releasing catheter and sustained-release catheter groups. (**a**) The blood concentration of rapamycin measured at 2 h, 12 h, 24 h, 72 h after a single oral dose (1.5 mg/kg), indwelling a rapamycin burst-releasing or sustained release catheter. (**b**) The blood concentration of rapamycin in the systemic administration group was measured every seven days, while those in the burst and sustained release catheter groups were below the lower limit of quantitation. LLQ: Lower Limit of Quantification. All data are presented as the Mean ± S.D. n=5 animals per group.

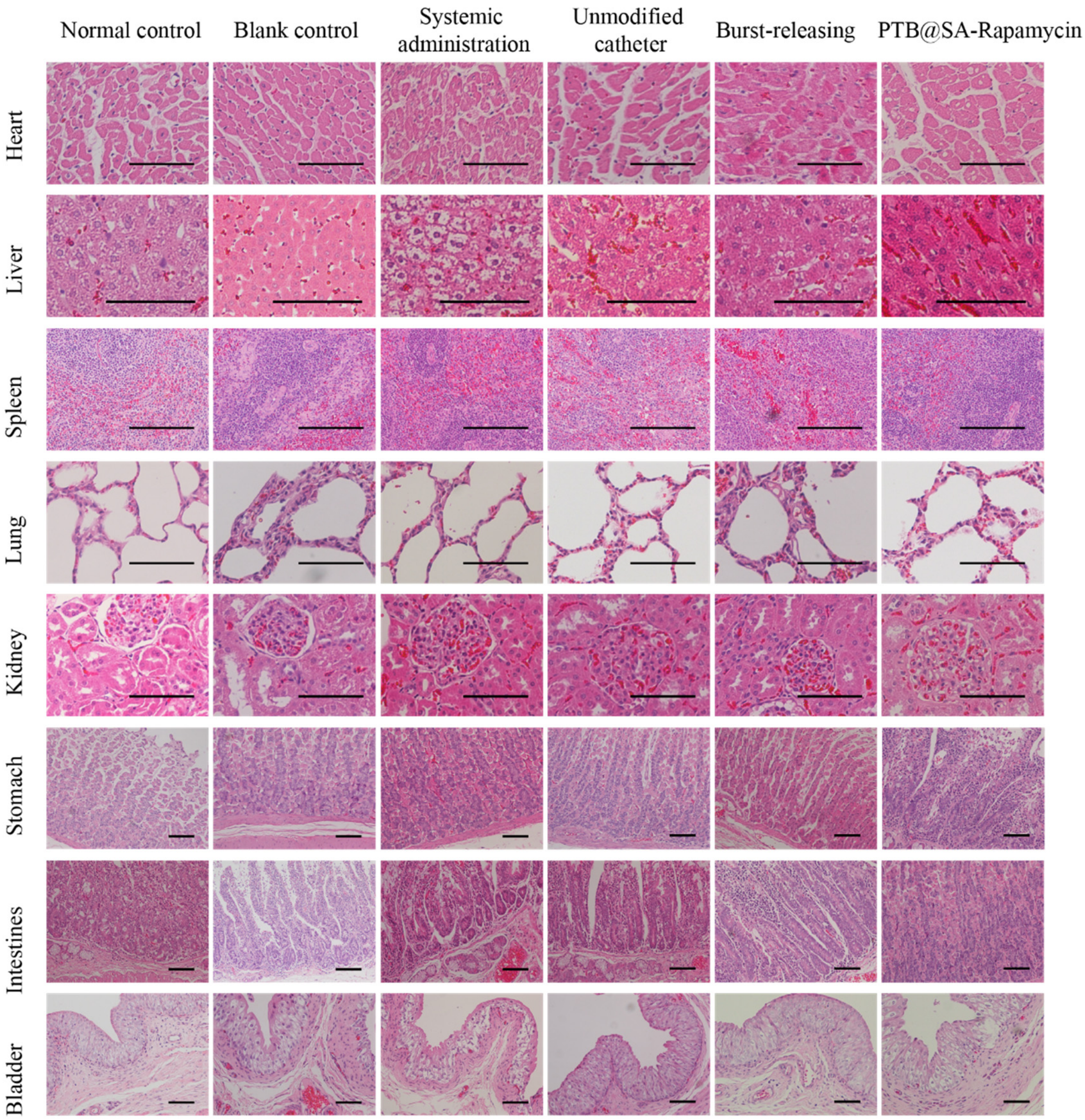

Supplementary Figure 43. H&E staining images of major organs for normal healthy group and rabbits in each group. After 30 days of different treatments, the major organs were collected and the tissue sections were stained with H&E. Compared with the healthy rabbit, the liver sections of the rabbits in the systemic administration group showed diffuse hepatocyte ballooning, which is a manifestation of liver cell damage, suggesting that the systemic spillover of rapamycin caused hepatotoxicity in rabbits. However, due to the limited systemic exposure of local administration, no damage was observed in rabbits treated with the PTB@SA-Rapamycin catheter, and the total drug amount of the sustained-release catheter was only 3 mg, whereas the cumulative intake of rapamycin in rabbits administered orally was as high as 135 mg. Scale bars, 100 μm. n=5 animals per group.

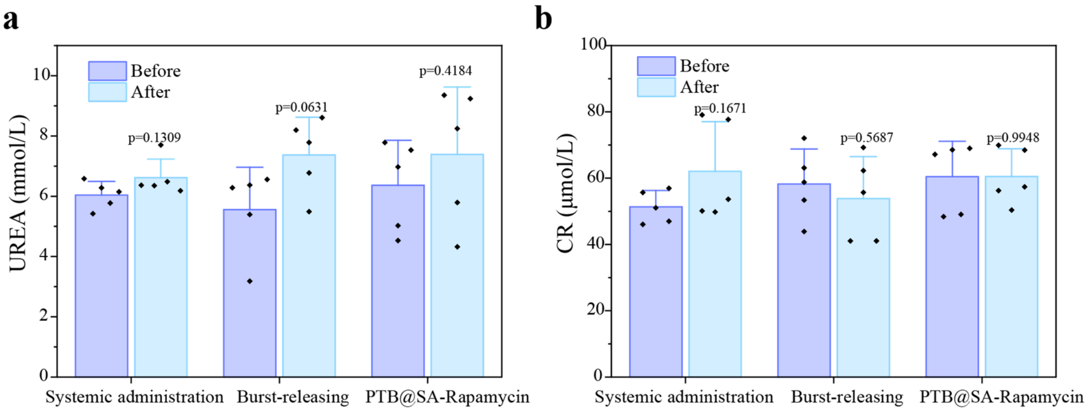

Supplementary Figure 44. Kidney function changes caused by systemic administration, indwelling burst-releasing or PTB@SA-Rapamycin catheter. Before and after the intervention, whole blood was collected and centrifuged at 840×g for 5 min to obtain serum for the determination of urea (**a**) and creatinine (CR, **b**). The data are presented as the Mean ± S.D. n=5 animals per group.

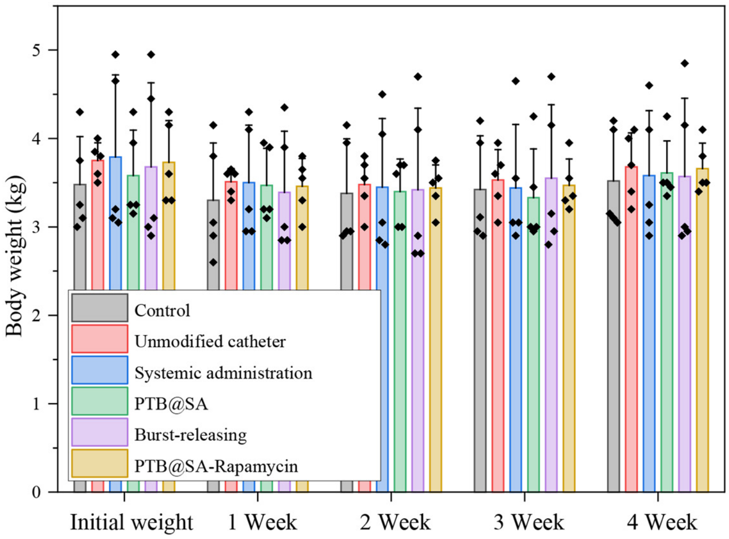

Supplementary Figure 45. The initial body weight and the weight of the rabbits in each group at 1, 2, 3 and 4 weeks after the urethra was injured by electrocoagulation. The data are presented as the Mean ± S.D. n=5 animals per group. At each time point, there was no statistical difference in body weight between the treatment groups and the blank control group. Statistics was calculated by two-way ANOVA followed by Dunnett's multiple comparisons test. Exact P values are given in the Source Data file.

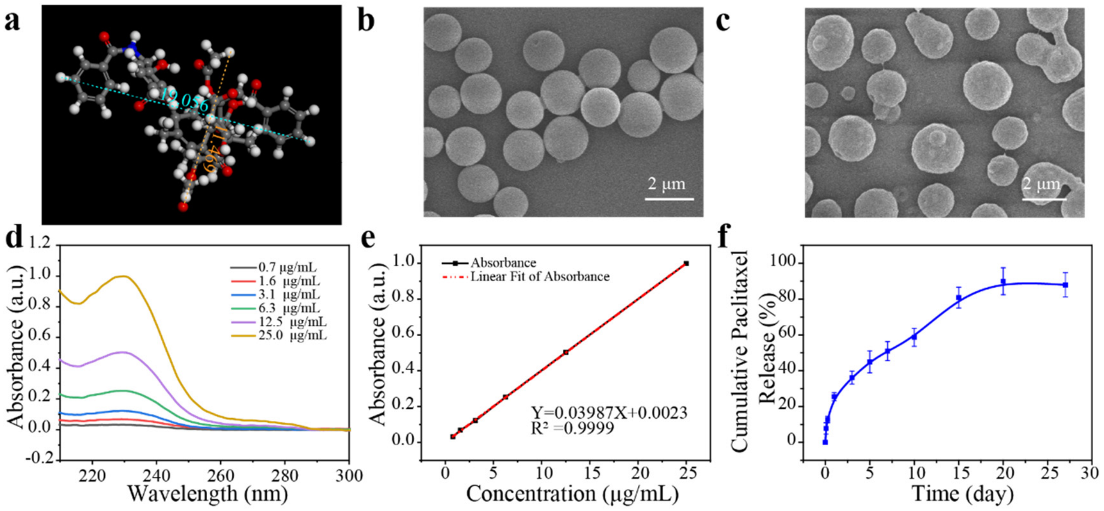

Supplementary Figure 46. The sustained release of paclitaxel controlled by the PTB@SA(0.10) nanofilm. (**a**) The structure and molecular size of paclitaxel depicted using BIOVIA Materials Studio 7.0 software. The unit of the molecular size shown in the inset is angstrom (Å). (**b**) SEM image showing bare paclitaxel particles on the PTB@SA(0.10) priming coating. (**c**) SEM image showing paclitaxel particles coated with the PTB@SA(0.10) nanofilm. (**d**) UV-Vis spectra of paclitaxel dissolved in release medium. (**e**) The standard curve and regression equation of paclitaxel in the release medium. (**f**) In vitro release behavior of paclitaxel controlled by the PTB@SA(0.10) nanofilm. Data are presented as the Mean ± S.D. n=6 independent samples per group.

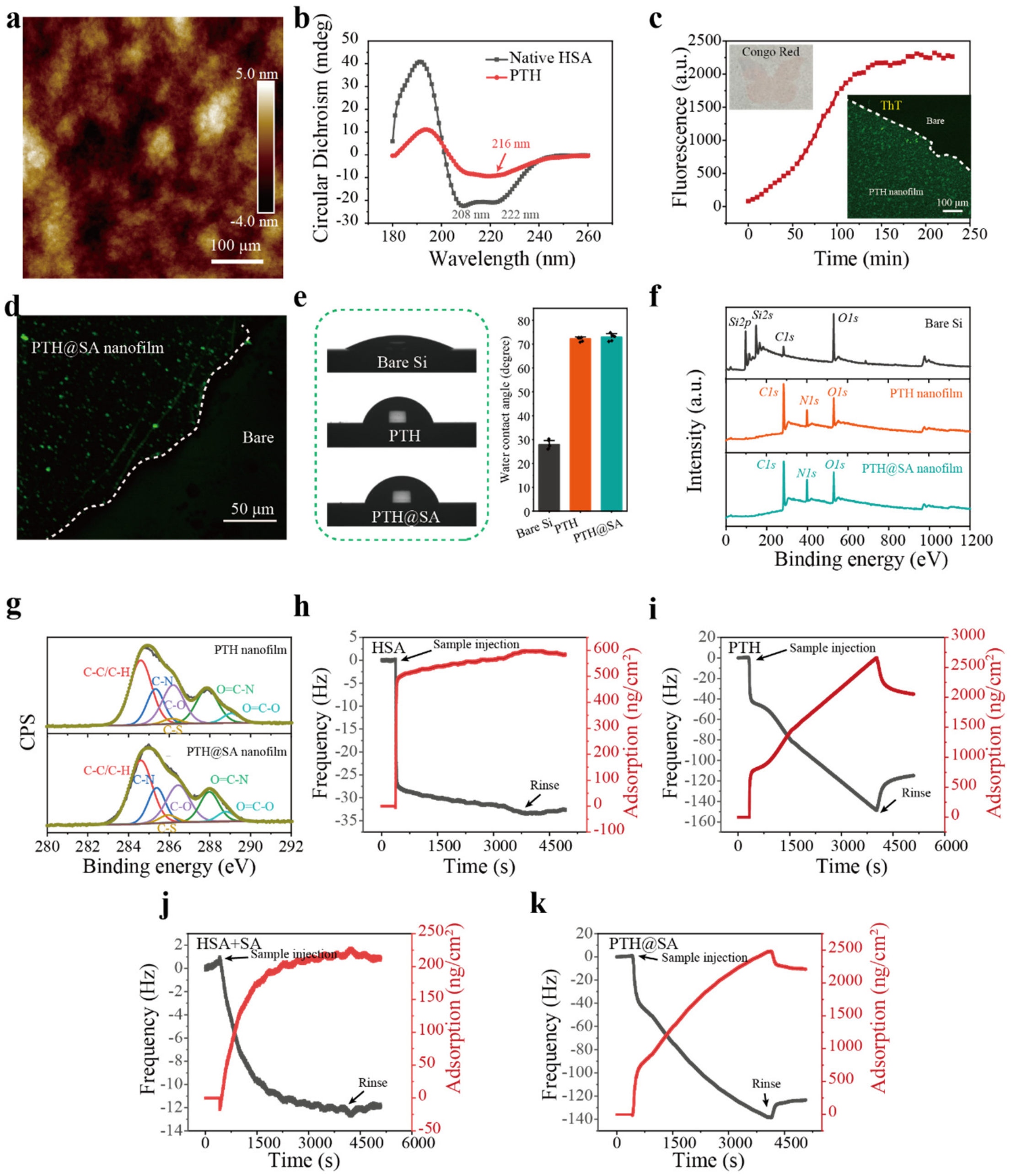

Supplementary Figure 47. Formation and hybridization of PTH the nanofilm. (**a**) AFM image of the PTH nanofilm. (**b**) CD spectra of native HSA and the PTH nanofilm. (**c**) ThT fluorescence change as a function of phase transition time, with the insets showing the corresponding fluorescence microscopic image for ThT staining and the optical microscopy image for Congo red staining. (**d**) The CLSM image shows that SA was successfully entrapped in the nanofilm to form a hybrid nanofilm. (**e**) WCA of bare Si, PTH and PTH@SA coated Si. Data are presented as the Mean ± S.D. n=6 independent samples per group. (**f**) XPS spectra of pristine Si, PTH and PTH@SA nanofilm coated Si. (**g**) High resolution *C1s* deconvolution spectra of the PTH and PTH@SA nanofilm. (**h-k**) The frequency and adsorption mass of HSA (**h**), PTH (**i**), the mixture of HSA and SA (**j**) and PTH@SA (**k**)adsorbed on the Au chip as a function of time.

Supplementary Discussion: As indicated by AFM, the PTH nanofilm was formed by close packing of nanoscale particles (Supplementary Fig. 47 a). Far-UV circular dichroism (CD) spectra confirmed the loss of α-helix (208 and 222 nm), accompanied by a significant increase in β-sheet (216 nm) in

the PTH nanofilm (Supplementary Fig. 47 b). The ThT fluorescence results of the phase transition system reflected the continuous accumulation of β-sheet structure in the reaction system, and the successful staining of the nanofilm with ThT and Congo red also indicated that the resultant nanofilm was rich in β-sheet (Supplementary Fig. 47 c). Based on the above, we further doped sodium alginate (SA) into the phase transition system and confirmed that SA was successfully integrated into the nanofilm (Supplementary Fig. 47 d). Compared with bare Si, both the water contact angle and surface elements changed significantly after incubating with the phase transition solution (Supplementary Fig. 47 e, f), indicating that the PTH or PTH@SA nanofilm was successfully coated on Si. The robust adhesion of the nanofilm originates from various polar or nonpolar functional groups exposed on the nanofilm surface that can interact with the underlying substrate in a variety of ways. As revealed by the high-resolution XPS spectra of *C1s* of the nanofilm (Supplementary Fig. 47 g), the nanofilm typically presented structures including aliphatic carbon (C-H/C-C), amines (C-N), hydroxyls (C-O), thiols (C-S) amides (O=C-N), and carboxyl groups (O=C-O). To further characterize the adsorption capacity of the samples, solutions with different components were pumped into the chamber of the QCM-D. After 1 h, the adsorption mass of the PTH and PTH@SA nanoparticles on the Au chip exceeded 2000 ng/cm^2, which was significantly higher than that of HSA and the mixture of HSA and SA (Supplementary Fig. 47 h-k). The above results indicate that HSA can also undergo a similar phase transition process triggered by TCEP, and the SA-doped polysaccharide-protein composite nanofilm can be easily obtained by adding SA to the phase transition system.

3. Supplementary Tables.

Supplementary Table 1. Drug release kinetics of rapamycin from a constant or nonconstant activity source.

Formulation	Model	Regression equation	R^2
Nonconstant activity source	First-order	In (1-Q) = -0.3477t	0.9902
	Zero-order	Q = 0.0465t + 0.3149	0.7794
Constant activity source	First-order	In (1-Q) = -0.1390t	0.9499
	Zero-order	Q = 0.0032t + 0.1985	0.9890

Supplementary Table 2. The calculated similarity factor (f_2) between compared formulations.

Compared formulations		Similarity factor (f_2)
Native BSA	Native BSA+SA	88.5
Native BSA	PTB	38.9
Native BSA+SA	PTB@SA(0.10)	30.4
PTB	PTB@SA(0.10)	34.5

Supplementary Table 3. Details of several typical commercially available rapamycin-eluting stents.

	Stent name	polymer	Coating method	Mechanism of controlled release	Design[a]	Refs
Permanent polymer	Cyper stent (Cordis Corporation, Bridgewater, NJ, USA)	PC, PEVA, PBMA	Base coat: PC; drug coat: PBMA, PEMA + Rapamycin; top coat: PBMA	diffusion	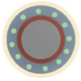	3
	FIREBIRD2 (MicroPort Medical, Shanghai, China)	Poly(styrene-butylene styrene)	Poly(styrene-butylene styrene) + Rapamycin	diffusion	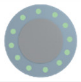	4
Biodegradable polymer	Supralimus (Sahajanand Medical Technologies, Pt. Ltd., India)	PLLA, PLGA, PLC, PVP	Base coat: PLLA, PLGA, PLC + Rapamycin; top coat: PVP	two layer preventing premature drug release	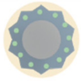	5
	Insprion (Scitech Medical, São, Paulo, Brazil)	PLLA, PDLLGA	Abluminal: PLLA, PDLLA + Rapamycin	hydrolysis and enzymatic action	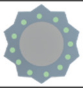	6
	NOYA (Medfavor Medical, Beijing, China)	PDLLA	PDLLA + Rapamycin	diffusion/degradation	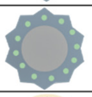	4
	Combo stent (Orbus Neich Medical, Hongkong, China)	SynBiosys: the degradation of the polymer	Abluminal: SynBiosys + Rapamycin; Luminal: CD34 antibody layer	hydrolysis/degradation	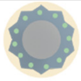	3

a. The gray represents the cross-section of a stent strut, the red color represents the base coat, the blue color represents the drug-containing matrix, the green color represents the anti-fibrotic drug, and the yellow color represents the top coat for slowing down the rate of drug release.

PC: parylene C; PEVA: poly(ethylene-co-vinyl acetate); PBMA: poly(n-butyl methacrylate); PLLA: poly(L-lactic acid); PLGA: poly(lactide-co-glycolide); PLC: poly(lactide-co-caprolactone); PVP: polyvinylpyrrolidone; PLLA: poly(L-lactic acid); PDLLGA: poly(D, L-lactide-coglycolide); PDLLA: poly(D, L -lactide).

Supplementary Table 4. Results of fitting the curves of in vitro release.

Formulation	Model	Regression equation	R^2
PTB	First-order	$\ln(1-Q) = -0.6010t$	0.8866
	Zero-order	$Q = 0.0016t + 0.3745$	0.6194
	Higuchi	$Q=0.0398t^{0.5} + 0.2157$	0.8479
	Biphasic model	$Q=1.1101-0.5211e^{-0.1797t}-0.5882e^{-0.0032t}$	0.9968
PTB@SA(0.10)	First-order	$\ln(1-Q) = -0.0024t$	0.9637
	Zero-order	$Q = 0.0009t + 1.1263$	0.9415
	Higuchi	$Q=0.0297t^{0.5} + 0.0056$	0.9950
	Biphasic model	$Q=1.4979-0.17281e^{-0.0755t}-1.3248e^{-0.0009t}$	0.9966

Supplementary Table 5. Overview of preclinical outcomes of topical therapy to prevent urethral strictures[7–10].

Study	Topical intervention	Lumen reduction		Mean percent improvements in lumen stenosis
		Control group	Experimental group	
Kurt 2017[7]	40 mg Triamcinolone, submucosal injection, once	91.0%	49.0%	46.2%
Chong 2011[8]	0.1 mg Rapamycin, urethral irrigation, daily for 28 days	69.1%	56.6%	18.1%
	1 mg Rapamycin, urethral irrigation, daily for 28 days	69.1%	36.5%	47.2%
Kurt 2017[7]	0.5 mg/mL Mitomycin-C (MMC), hydropathic compress, once	91.0%	45.0%	50.5%
Shinchi 2019[9]	0.2 mg Insulin-like growth factor 1 (IGF-1), impregnated collagen sutured to the catheter, catheterization for 14 days	79.8%	39.3%	50.8%
Fu 2014[10]	0.01 mg Docetaxel, urethral irrigation, daily for 28 days	84.7%	48.0%	43.3%
	0.1 mg Docetaxel, urethral irrigation, daily for 28 days	84.7%	36.9%	56.4%
This research	3 mg Rapamycin, sustained release coating modified catheter, catheterization for 30 days	51.8%	10.8%	79.2%

4. Supplementary References.

1. Koster, R. A., Dijkers, E. C. F. & Uges, D. R. A. Robust, high-throughput LC-MS/MS method for therapeutic drug monitoring of cyclosporine, tacrolimus, everolimus, and sirolimus in whole blood. *Ther. Drug Monit.* **31**, 116–125 (2009).
2. Jones, D. S., Djokic, J. & Gorman, S. P. Characterization and optimization of experimental variables within a reproducible bladder encrustation model and in vitro evaluation of the efficacy of urease inhibitors for the prevention of medical device-related encrustation. *J. Biomed. Mater. Res. - Part B Appl. Biomater.* **76**, 1–7 (2006).
3. Granada, J. F. *et al.* Development of a novel prohealing stent designed to deliver sirolimus from a biodegradable abluminal matrix. *Circ. Cardiovasc. Interv.* **3**, 257–266 (2010).
4. Xu, B. *et al.* Nine-month angiographic and 2-year clinical follow-up of the NOYA biodegradable polymer sirolimus-eluting stent in the treatment of patients with de novo native coronary artery lesions: The NOYA I trial. *EuroIntervention* **8**, 796–802 (2012).
5. Dani, S. *et al.* Biodegradable-polymer-based, sirolimus-eluting Supralimus® stent: 6-month angiographic and 30-month clinical follow-up results from the Series I prospective study. *EuroIntervention* **4**, 59–63 (2008).
6. Ribeiro, E. E. *et al.* First-in-man randomised comparison of a novel sirolimus-eluting stent with abluminal biodegradable polymer and thin-strut cobalt-chromium alloy: INSPIRON-I trial. *EuroIntervention* **9**, 1380–1384 (2014).
7. Kurt, O. *et al.* Effect of mitomycin - C and triamcinolone on preventing urethral strictures. *Int. Braz J Urol* **43**, 939–945 (2017).
8. Chong, T. *et al.* Rapamycin inhibits formation of urethral stricture in rabbits. *J. Pharmacol. Exp. Ther.* **338**, 47–52 (2011).
9. Shinchi, M. *et al.* Insulin-like growth factor 1 sustained-release collagen on urethral catheter prevents stricture after urethral injury in a rabbit model. *Int. J. Urol.* **26**, 572–577 (2019).
10. Fu, D., Chong, T., Li, H., Zhang, H. & Wang, Z. Docetaxel inhibits urethral stricture formation, an initial study in rabbit model. *PLoS One* **9**, 1–6 (2014).
